# The Interpeduncular-Ventral Hippocampus Pathway Mediates Active Stress Coping and Natural Reward

**DOI:** 10.1523/ENEURO.0191-20.2020

**Published:** 2020-11-16

**Authors:** Yasmine Sherafat, Malia Bautista, J. P. Fowler, Edison Chen, Amina Ahmed, Christie D. Fowler

**Affiliations:** Department of Neurobiology and Behavior, University of California Irvine, Irvine, CA 92697

**Keywords:** anhedonia-associated behavior, hippocampus, interpeduncular nucleus, serotonin, stress coping behavior

## Abstract

Maladaptive stress-related behaviors are integral to multiple complex psychiatric disorders, and it has been well established that serotonergic signaling mediates various aspects of these maladaptive states. In these studies, we sought to uncover the function of a previously undefined serotonergic pathway, which projects from the interpeduncular nucleus (IPN) to the ventral hippocampus (vHipp). Intersectional retrograde and chemogenetic viral manipulation strategies were employed to manipulate the function of the IPN-vHipp pathway during a variety of behavioral measures in male mice. We found a significant effect of circuit inhibition on behaviors associated with coping strategies and natural reward. Specifically, inhibition of the IPN-vHipp pathway dramatically increased active stress-induced escape behaviors, in addition to moderately affecting sucrose consumption and food self-administration. During inhibition of this pathway, agonist activation of serotonergic 5-HT_2A/2C_ receptors in the vHipp reversed the effects of IPN-vHipp circuit inhibition on active escape behaviors, thereby supporting the synaptic mechanism underlying the behavioral effects evidenced. IPN-vHipp inhibition did not induce differences in generalized locomotion, anxiety-associated behavior, and intravenous nicotine self-administration. Importantly, these findings are in opposition to the canonical understanding of serotonin in such escape behaviors, indicating that serotonin exerts opposing effects on behavior in a pathway-specific manner in the brain. Taken together, these findings thereby have important implications for our understanding of serotonergic signaling and associated therapeutic approaches for the treatment of disease symptomology.

## Significance Statement

Deficits in serotonergic signaling are associated with depression-associated behaviors, such as a reduction in escape behaviors (e.g., learned helplessness) and anhedonia, whereas global pharmacological approaches that increase synaptic serotonin, such as SSRIs, ameliorate these behaviors in animal models. In these studies, we found that inhibition of a previously undefined pathway, consisting of serotonergic projections from the interpeduncular nucleus (IPN) to the ventral hippocampus (vHipp), increases active stress-induced escape behaviors, food self-administration, and natural reward consumption. Importantly, these findings define the function of this novel pathway, and, in doing so, provide evidence that decreased serotonergic signaling in this pathway leads to excessive active escape behaviors under stress conditions, which may represent symptoms associated with the pathologic state.

## Introduction

Functionally, the serotonergic system has been shown to underlie symptomology associated with complex psychiatric disorders. As one example, major depressive disorder is characterized by anhedonia, feelings of worthlessness, maladaptive stress response, and thoughts of suicide in humans (Earl, 2017). Patients with depressive symptoms have been found to exhibit abnormalities in brain serotonergic signaling mechanisms (Young et al., 1985; Drevets et al., 1999). The first-line treatments for symptomology associated with depression are selective serotonin reuptake inhibitors (SSRIs), which act by increasing serotonin levels in the synapse (Young et al., 1985; Pundiak et al., 2008). While SSRIs are found to be efficacious for many individuals, limitations have been noted in their therapeutic effectiveness (Nestler et al., 2002). For instance, SSRIs act immediately to increase synaptic serotonin, but patients often do not report beneficial effects until after many weeks of treatment (Nestler et al., 2002; Blier, 2009). In addition, during the initial treatment period, an increase in suicidal risk has been reported, especially among males (Barbui et al., 2009; Coupland et al., 2015), suggesting that the underlying serotonergic mechanisms are not fully understood with such global pharmacological approaches. Therefore, a more systematically defined understanding of the neural circuits that mediate states associated with stress-related symptomology is needed.

Neurons in the raphe nuclei are primarily responsible for releasing serotonin throughout the brain (Liu et al., 2002; Abela et al., 2020). In addition to synthesizing serotonin, these cells express multiple serotonergic receptor subtypes, which may function at both the postsynaptic and presynaptic membranes (Barabanova et al., 2007; Mezadri et al., 2011). Presynaptic receptors have been shown to modulate release of neurotransmitters, such as glutamate and serotonin (Pinheiro and Mulle, 2008; Stahl, 2015). Interestingly, a discrete population of neurons outside of the raphe nuclei have also been identified as expressing serotonin (Groenewegen and Steinbusch, 1984). These neurons are localized in the interpeduncular nucleus (IPN) and project to the ventral hippocampus (vHipp; Groenewegen and Steinbusch, 1984). Based on the IPN’s afferent and efferent connections within the limbic system, the IPN has been characterized as a signal integration center and has been implicated in a wide variety of functions, including nicotine reinforcement, aversion and withdrawal, sleep regulation and pain sensitivity (Mészáros et al., 1985; Haun et al., 1992; Fowler et al., 2011; Zhao-Shea et al., 2013; Ables et al., 2017; Tuesta et al., 2017; Wolfman et al., 2018; Arvin et al., 2019; Antolin-Fontes et al., 2020). The vHipp has been mainly implicated in anxiety, avoidance behaviors, and contextual fear learning (Kenney et al., 2012; Çalışkan and Stork, 2019; Hjorth et al., 2019; Padilla-Coreano et al., 2019). Furthermore, in humans, smaller hippocampal volumes in major depressive disorder are linked to more severe symptoms, early age onset, and non-responsiveness to treatment (Vakili et al., 2000; Sheline et al., 2003; Frodl et al., 2004; Lloyd et al., 2004), suggesting that the hippocampus may be involved in various aspects of disease symptomology. Although the IPN-vHipp pathway has been previously identified (Groenewegen and Steinbusch, 1984), the function of this circuit had remained elusive.

In these studies, we sought to investigate the IPN-vHipp pathway using an intersectional viral manipulation strategy in mice. Animal models allow for the discrete dissection of circuit function and can thus reveal signaling mechanisms underlying symptoms associated with psychiatric disorders. Recently, research domain criteria (RDoC) have been proposed as a means of classifying and studying behavioral subsets that contribute to such symptomology (RDoC Initiative, 2020). Thus, given the unknown function of this circuit, mice in these studies were examined in a range of behavioral assessments, including nicotine reinforcement, motivated behavior to obtain food reward, anxiety-associated behavior, generalized locomotion, reward/aversion conditioned place preference, natural reward consumption, and stress-induced coping behavior. We found that the IPN-vHipp pathway is specifically involved in mediating active coping under stress conditions and natural reward. Specifically, inhibition of the IPN-vHipp circuit increased active escape behaviors, sucrose consumption, and food reinforcement during. Since the IPN neurons were found to express serotonergic markers, we used a site-specific pharmacological approach to further establish that 5-HT_2A/2C_ receptors in the vHipp mediate the pronounced effects on stress-induced escape behavior.

## Materials and Methods

### Mice

Male wild-type C57BL/6J mice were obtained from the Jackson Laboratory (catalog #000664). For initial track tracing studies to visualize cre expression with retrograde viral injection, ROSA^26Sor^-tdTomato reporter mice were obtained from The Jackson Laboratory (strain B6.Cg-Gt(ROSA)26Sortm14(CAG-tdTomato)Hze/J;https://www.jax.org/strain/007914; RRID:IMSR_JAX:007914). All mice were at least six weeks of age at the beginning of the experiments and were housed in a humidity and temperature-controlled (22°C) vivarium on a reverse 12/12 h light/dark cycle. For all behavioral analyses, mice were habituated to the rooms and experimenters across 2 d prior, and all assessments were scored by experimenters blinded to the group/injection conditions. For all of the mice receiving repeated injections, the clozapine N-oxide (CNO) or vehicle injections were administered with a minimum of 3 d apart to allow for a wash-out period. All procedures were conducted in strict accordance with the NIH *Guide for the Care and Use of Laboratory Animals* and were approved by the Institutional Animal Care and Use Committee of the University of California, Irvine.

Mice used in these studies included wild-type and Rosa-tdTomato mice (*n* = 4) for initial circuit tracing of the IPN-vHipp circuit. Experimental mice included six sets expressing a cre-dependent Designer Receptors Exclusively Activated by Designer Drugs (DREADD) AAV-hM4Di and retrograde AAV-cre in the IPN-vHipp circuit as follows:

1. An initial cohort (*n* = 4) were first examined for stress coping behavior with a between-subject design, and thereafter, these mice were perfused to validate specific localization of the virus expression. After virus expression was confirmed, we proceeded to test subsequent mice (*n* = 8) for stress coping behavior (between-subject design). Thereafter, these eight mice were also tested for sucrose consumption (within-subject cross-over), and then an open field locomotion test (between subject design).
2. Given that the initial locomotor assessment had high variability with the low subject number, we included an additional set of mice (*n* = 14, between-subject design) that were only tested in the open field (e.g., without any prior CNO exposure or behavioral assessments).
3. The third set of mice (*n* = 7, within-subject design) were examined in the elevated plus maze (EPM), followed by conditioned place preference, followed by food self-administration, and then intravenous nicotine self-administration (0.03 mg/kg/infusion acquisition dose and then 0.4 mg/kg/infusion high dose). One mouse did not survive the intravenous surgical procedure, and thus was not included in the nicotine self-administration part of the study.
4. The fourth set of mice (*n* = 11, between-subject design) were analyzed for c-fos expression in the hM4Di-expressing IPN neurons.
5. The fifth set of mice (*n* = 11, between-subject design) were cannulated and examined in the stress coping behavioral assessment following 2,5-dimethoxy-4-iodoamphetamine (DOI) or vehicle microinjections in the vHipp.
6. A final set of mice (*n* = 9) were cannulated and examined in the stress coping test for additional control conditions [e.g., vehicle peripheral injections (between subject factor) and either local microinfusion of saline or DOI (within subject factor)]. Thereafter, these mice were examined for the effects of the injections in the locomotor assessment. Finally, 4 of these mice were randomly selected as an additional cohort to validate replication of the findings in the sucrose study, using a between subject design.

AAV-control vector and retrograde AAV-cre-injected mice (*n* = 14, between-subject design) were included to examine the effects of CNO alone in the stress coping behavioral assessment. For these 14 total mice, the initial cohort (*n* = 3) were perfused and examined for specific localization of the control vector virus expression immediately following the stress coping behavioral assessment. The remaining 11 mice were subject to further analysis for sucrose consumption after a minimum 3-d wash-out period with a between-subject design. In consideration of recent studies that found that CNO back metabolizes to clozapine (Manvich et al., 2018), these additional controls were necessary to support the experimental findings.

### Drugs

CNO (catalog #16882, Caymen Chemicals) was dissolved in vehicle, which consisted of 0.1% dimethylsulfoxide in 99.9% sterile saline solution. Subjects were injected subcutaneously with vehicle or CNO (3 mg/kg) at an injection volume of 10 ml/kg and placed back into the home cage for 20 min before each behavioral assessment. This CNO dose and injection site were selected based on prior reports (Marchant et al., 2016; Padovan-Hernandez and Knackstedt, 2018; Mahler et al., 2019; Guarino et al., 2020), and given that a previous pharmacokinetic study demonstrated that a 3.5 mg/kg subcutaneous injection of CNO in mice increased levels of CNO in CSF and total brain concentration above EC_50_ for 15–60 min after injection (Jendryka et al., 2019). For site-specific brain injections, DOI (catalog #2643, Tocris) was dissolved in saline vehicle and microinjected into the vHipp through the bilateral guide cannula (0.5 μg/0.5 μl injection per side) across 2 min in the home cage; the injector remained in place for at least three additional minutes to allow for diffusion before removal.

### Stereotaxic AAV injections and cannulations

Subjects were anesthetized with a 1–3% isoflurane/oxygen mixture and positioned in a Kopf stereotaxic frame with the incisor bar set to the flat-skull position. Brain microinjections were administered into the IPN or bilaterally into the vHipp at a volume of 0.5 or 0.25 μl, respectively, at a rate of 0.25 μl/min. Following each infusion, the injector remained in place for an additional 5–9 min. The coordinates were as follows: rostral IPN, midline: 10° angle toward midline, AP −3.60 mm, ML: ±0.8 mm, DV −4.20 mm; vHipp, bilateral: 0° angle, AP −2.92 mm, ML: ±3.10 mm, DV −2.9 mm. Initial track tracing was conducted with the retrograde tracer Fluoro-Gold (Flurochrome Inc; Catapano et al., 2008). For experimental groups, viral vectors included: AAV-hSyn.DIO.hM4D(Gi).mCherry (catalog #44362-AAV8, Addgene; IPN injection), AAV-hSyn.DIO.mCherry (control AAV vector; catalog #50459-AAV8, Addgene; IPN injection), AAVrg-pENN.AAV.hSyn.HI.eGFP-Cre.WPRE.SV40 (catalog #105540-AAVrg, Addgene; vHipp injection), and AAVrg-pENN.AAV.hSyn.Cre.WPRE.hGH (catalog #105553-AAVrg, Addgene; vHipp injection). For the site-specific drug injections, mice were cannulated. After the DREADD virus injection into the IPN, mice were implanted bilaterally with double guide cannula into the vHipp (AP: −2.92 mm, ML: ±3.10 mm, DV: −1.9 mm); retrograde virus injections were administered 1 mm below the tip of each cannula, and during the behavioral testing, DOI or saline was administered to the same hippocampal location via injectors placed in the cannula. At the end of the study, virus expression and cannula placement were validated for all subjects. Coordinates were based on the mouse brain atlas (Paxinos and Franklin, 2001) and as determined from previous injection and tracer studies. Mice were permitted to recover for three weeks to allow for viral expression before any behavioral assessments.

AAV Viral preps for the above were obtained from Addgene. The provider source information is as follows for each plasmid. The pAAV-hSyn-DIO-hM4D(Gi)-mCherry was a gift from Bryan Roth (Addgene viral prep #44362-AAV8; http://n2t.net/addgene:44362; RRID:Addgene_44362). The pAAV-hSyn-DIO-mCherry was a gift from Bryan Roth (Addgene viral prep #50459-AAV8; http://n2t.net/addgene:50459; RRID:Addgene_50459). The pENN.AAV.hSyn.HI.eGFP-Cre.WPRE.SV40 was a gift from James M. Wilson (Addgene viral prep #105540-AAVrg; http://n2t.net/addgene:105540; RRID:Addgene_105540). The pENN.AAV.hSyn.Cre.WPRE.hGH was a gift from James M. Wilson (Addgene viral prep #105553-AAVrg; http://n2t.net/addgene:105553; RRID:Addgene_105553).

### Stress coping behavioral assessment

For this study, we sought to induce a state of mild stress to examine stress coping behaviors, while allowing for a relative increase or decrease in behavioral output (Fitzgerald et al., 2019). Thus, mice were socially isolated after AAV injections for three weeks before the behavioral assessment (Kalliokoski et al., 2014). Thereafter, subjects were tested for stress coping behaviors in the forced swim water chamber as described previously (Pushkin et al., 2019). This assessment was employed to examine the RDoC domain of Negative Valence Systems (responses to aversive situations, including fear) with the constructs of acute threat (perceived danger displayed in pattern of adaptive responses), potential threat (behavioral responses for enhanced risk assessment), and frustrative non-reward (inability to escape chamber despite sustained escape attempts; RDoC Initiative, 2020). Further RDoC domains required for mice to perform this task include the domain of cognitive systems with the constructs of perception (multimodal somatosensory perception), cognitive control (response selection, inhibition/suppression), and sensorimotor systems (innate motor patterns; RDoC Initiative, 2020). For this test, a cylindrical glass chamber (height 21 cm, diameter 19 cm) was filled with room temperature water (22−23°C) at a water depth of 12 cm. On the testing day, mice were subcutaneously administered CNO or vehicle, placed back into the home cage for 20 min, and then placed in the water chamber for the stress coping assessment test. Each test was 5 min in duration and video recorded. For the site-specific brain microinjections, mice were first injected with CNO or vehicle subcutaneously, and placed back into the home cage for 15 min. Thereafter, they were gently restrained and microinjected with DOI (0.5 µg/0.5 µl injection bilaterally) or saline vehicle through the guide cannula for a 5-min duration, and then placed back into the home cage for 10 min before the stress coping assessment. Given that mice may habituate with repeated testing in this assessment, a between-subject experimental design was employed. Time immobile and number of immobile bouts were scored by experimenters blinded to the group conditions.

### Natural reward consumption

Mice were examined for their level of sucrose consumption under full food conditions (e.g., no food restriction), which has been suggested to represent a measure of behavior associated with an anhedonia state for low levels of consumption (Monleon et al., 1995). This was of particular interest for these investigations given the role of the IPN in mediating satiety signals (Tuesta et al., 2017). This assessment represents the RDoC domain of positive valence systems, with the construct of reward responsiveness and subconstruct of reward satiation for consummatory behavior (RDoC Initiative, 2020). Subjects were first habituated to sucrose pellets in the home cage 2 d before the test day (60 mg per mouse; 5TUT, raspberry-flavored, TestDiet). On the test day, mice were subcutaneously injected with CNO or vehicle and placed back in the home cage for 20 min. Thereafter, they were individually placed in a standard home cage (empty clean cage) and provided 400 mg of sucrose pellets for 1 h. Sucrose pellet consumption was scored by experimenters blinded to the group conditions.

### Conditioned place preference

The conditioned place preference paradigm allows for the assessment of both reward-associated and aversion-associated learning processes, in which a subject chooses to spend time in the chamber previously associated with reward or aversion, respectively. This test was employed to determine if inhibition of this circuit alone was sufficient to induce a rewarding or aversive state. Given the unknown function of the IPN-vHipp circuit, either outcome was hypothesized as plausible. This assessment corresponds to the RDoC domains of positive valence systems with the construct of reward learning and subconstruct of probabilistic learning (environmental stimuli associated with a reinforcer), and negative valence systems with the construct of acute threat (conditioned stimuli; RDoC Initiative, 2020). Further, the cognitive systems RDoC domain was also assessed with subconstructs of perception and declarative memory (RDoC Initiative, 2020). Studies were conducted using a three-compartment apparatus with two equal-sized chambers (17 × 12.7 × 12.3 cm) separated by a neutral center chamber (8.5 × 12.7 × 12.3 cm). The large compartments differed in the wall stripes (horizontal or vertical) and flooring (smooth or small holes) and were separated from the center compartment by sliding doors. Mice were first assessed for baseline preference across a 15-min session, in which each animal was placed in the center chamber and then permitted to freely move throughout the apparatus. Thereafter, mice were randomly assigned into drug injection conditions, in which each chamber and injection pairing was assigned in a counterbalanced manner. Mice were subsequently conditioned across six consecutive days with alternating CNO or vehicle sessions. For each session, animals were injected with CNO or vehicle 20 min before being confined to the assigned chamber. On the test day, mice were placed in the center chamber and then were permitted to freely move throughout the apparatus for 15 min. The time spent in each chamber was video recorded and scored with ANY-maze software by experimenters blinded to the injection/chamber pairing conditions.

### Open field locomotor behavior

Subjects were examined in an open field chamber for generalized locomotor behavior during a 15-min test as described previously (Chen et al., 2018). This important control condition was included to ensure that the differences found in the active escape behavior assessments were not secondary to differences in generalized locomotion during CNO-mediated circuit inhibition; specifically, this assessment corresponds to the RDoC domain of sensorimotor systems with the constructs of motor actions and innate motor patterns (RDoC Initiative, 2020). Briefly, the chamber was composed of Plexiglas (35 cm long × 35 cm wide × 31 cm high) and illuminated by a lamp for consistent lighting. The center and outer edge zones were designated with ANY-maze computer software during video analysis. On the test day, mice were injected with CNO or vehicle, placed back into the home cage for 20 min, and then placed into the center of the open field apparatus for the 15-min test. For the DOI study, mice were first subcutaneously injected with CNO or vehicle, and placed back into the home cage for 15 min; thereafter, they were microinjected with either DOI or saline for a 5-min duration, followed by being placed in the home cage for 10 min, and then they were placed in the open field for testing. Activity was recorded with a video camera, and distance traveled was scored with ANY-maze software.

### Elevated plus maze (EPM)

Subjects were examined for anxiety-related behavior in the EPM during a 5-min test as previously described (Chen et al., 2018). This assessment was employed to further assess the role of the IPN-vHipp circuit in the RDoC domain of negative valence systems with the construct of potential threat (anxiety; RDoC Initiative, 2020). The EPM was composed of four opaque gray runways 5 cm wide and 35 cm in length, which were elevated 40 cm from the floor. Two opposing closed runways had opaque walls 15 cm in height (closed arms), whereas the other two opposing sides did not contain walls (open arms). A shielded lamp was placed above the center of the maze for consistent lighting. Mice were injected with CNO or vehicle, placed back into the home cage for 20 min, and then placed in the center portion of the EPM with their head facing into an open arm of the maze. Subsequent behavior was recorded for 5 min thereafter with a video camera. Subjects were examined with a within-subject design, in which a minimum of 5 d was imposed in between testing sessions, and CNO or vehicle injections were administered in a crossover design. Time spent in each arm was scored with ANY-maze software, in which the animal’s head was used as the designated point to quantify entry and duration in an arm.

### Food and intravenous nicotine self-administration

To examine whether the IPN-vHipp pathway was involved in reinforcement for food or nicotine, mice were tested in the operant self-administration procedure. These assessments correspond to the RDoC constructs of positive valence systems with constructs of reward responsiveness (subconstructs of response to reward and reward satiation), reward learning (subconstructs of reinforcement learning and habit) and reward valuation (subconstructs of reward and delay), and Sensorimotor Systems with constructs of motor actions and habit (RDoC Initiative, 2020). For these assessments, mice were mildly food restricted to 85–90% of their free-feeding body weight and trained to press a lever in an operant chamber (Med Associates) for food pellets (5TUM, TestDiet) under a fixed-ratio 5, time out 20 s (FR5TO20 s) schedule of reinforcement. Upon completion of five lever presses, the food pellet is provided in the hopper and a cue light illuminates above the active lever for the duration of the 20-s time-out period. Once stable responding was achieved (>30 pellets per session across three subsequent sessions), subjects were administered CNO or vehicle 20 min before the session in a counterbalanced crossover design, with baseline days in between each CNO (or vehicle) injection. Thereafter, mice were surgically catheterized as previously described (Fowler and Kenny, 2011; Chen et al., 2018). Briefly, mice were anesthetized with an isoflurane (1–3%)/oxygen vapor mixture and prepared with intravenous catheters. The catheter tubing was passed subcutaneously from the animal’s back to the right jugular vein, and a 1-cm length of the catheter tip was inserted into the vein and tied with surgical silk suture. One mouse did not survive the surgical procedure and was thus excluded from the nicotine studies. Following the surgical procedure, animals were allowed ≥72 h to recover from surgery, then provided access to respond for food reward. Subjects were then permitted to acquire intravenous nicotine self-administration during 1 h daily sessions, 6 d per week, at the standard training dose of nicotine (0.03 mg/kg/infusion). Nicotine was delivered through tubing into the intravenous catheter by a Razel syringe pump (Med Associates). Each session was performed using two retractable levers (one active, one inactive). Completion of the response criteria on the active lever resulted in the delivery of an intravenous nicotine infusion and cue light (0.03-ml infusion volume; FR5TO20 s schedule). Responses on the inactive lever were recorded but had no scheduled consequences. Catheters were flushed daily with physiological sterile saline solution (0.9% w/v) containing heparin (100 USP units/ml). Catheter integrity was tested with Brevital (methohexital sodium, Eli Lilly). After establishing baseline responding across 8 d at the 0.03 mg/kg infusion dose, subjects were tested for differences in responding following CNO or vehicle administration. Injections were administered in a counterbalanced manner 20 min before self-administration sessions, and subjects were provided at least three baseline days in between each CNO (or vehicle) injection for within-subject testing. To examine whether responses differed on a higher dose, mice were transitioned subsequently onto the 0.4 mg/kg/infusion dose, and after establishing baseline responding, they were then injected with CNO or vehicle before the sessions as described above. Behavioral responses were automatically recorded by Med Associates software.

### Immunohistochemistry and RNAscope brain tissue analysis

Brain tissue was examined to confirm the IPN-vHipp circuitry, the effects of hM4Di inhibition on cellular activation in the IPN, and serotonin receptor expression in this pathway. All subjects were deeply anesthetized with ketamine-xylazine and perfused through the ascending aorta with saline (0.9% w/v) followed by 4% paraformaldehyde in 0.1 m PBS, pH 7.4. Thereafter, brains were removed and postfixed for 2 h in paraformaldehyde, followed by cryoprotection in 30% sucrose for >72 h. Brain sections were cut on a cryostat at 35-µm intervals. First, to validate the stereotaxic coordinates and serotonergic circuit, we examined brain tissue from Rosa-tdTomato reporter mice. This allowed us to verify the localization of cell bodies in the IPN that project to the vHipp. Specifically, Rosa-tdTomato reporter mice were injected with the retrograde cre-expressing AAV in the vHipp and thereafter were permitted three weeks for viral expression before perfusion. IPN-containing sections were processed for immunolabeling with 1:500 rabbit anti-serotonin (Immunostar, catalog #20080) and 1:1000 chicken anti-mCherry (Abcam, catalog #ab205402) diluted in 0.5% Triton X-100 in 0.1 m PBS (0.5% PBT) with 10% normal donkey serum (NDS) overnight at 4°C. After rinsing, sections were then incubated in 1:400 dilution of the secondary antibodies Alexa Fluor 488 donkey anti-rabbit and Alexa Fluor 594 donkey anti-chicken, followed by rinsing and mounting onto microscope slides. Sections were then coverslipped with Vectashield containing DAPI (Vector Labs, catalog #H-1200), and slides were examined with a Leica DM4000 fluorescence microscope.

To assess hM4Di DREADD-mediated changes in cellular activation during the active coping behavioral assessment, mice expressing the DREADD AAV in the IPN and retrograde AAV-Cre in the vHipp were injected with either vehicle or CNO 20 min before the start of the session. Subjects were then perfused 1.5 h thereafter. Sections were processed for immunolabeling using 1:1000 chicken anti-mCherry (Abcam, catalog #ab205402) and 1:1000 rabbit anti-c-fos (Abcam, catalog #ab190289) diluted in 0.5% PBT with 10% NDS overnight at 4°C. After rinsing, sections were then incubated in 1:400 Alexa Fluor 488 donkey anti-chicken and Alexa Fluor 647 donkey anti-rabbit secondary antibodies diluted in 0.5% PBT for 2 h at room temperature. Sections were then rinsed, mounted and coverslipped with Vectashield containing DAPI. Slides were examined with a Leica DM4000 fluorescence microscope with the same 40× magnification, gain, and exposure levels across subjects/groups. Colocalization of c-fos and mCherry labeled cells was scored manually by experimenters blinded to the group conditions. Finally, to examine the serotonin 2C (5-HT_2C_) receptor subtype expression in the mCherry DREADD-expressing IPN-vHipp neurons, sections were processed for RNAscope Multiplex Fluorescent assay (Advanced Cell Diagnostics). Briefly, sections were placed in an incubator for 30 min at 60°C then treated at 100°C for 6 min in target retrieval solution. Sections were dehydrated in 100% ethanol and treated with protease (Advanced Cell Diagnostics, catalog #322380). RNA hybridization probes included *fos* (Advanced Cell Diagnostics, catalog #555071-C1), *mCherry* (Advanced Cell Diagnostics, catalog #431201-C2), and *5htr2c* (Advanced Cell Diagnostics, catalog #401001-C3), which were labeled with Opal 520, Opal 570, and Opal 690 (PerkinElmer), respectively. Slides were then counterstained and coverslipped with Vectashield containing DAPI (Vector Laboratories) and imaged with a Leica SP8 confocal microscope at 63× magnification.

### Approach for unbiased data collection

All data were collected within each experiment by experimenters blinded to the testing condition. The results were then sent to another investigator not involved in the experimental analysis for decoding and statistical analyses. Each behavior was scored by two blinded experimenters to provide further confidence in the findings. When possible, Med Associates and ANY-maze computer software were used to quantify data points to ensure objective behavioral assessments.

### Statistical analysis

We used an experimental design with random assignment. Data were analyzed using estimation statistics (Ho et al., 2019) via the website analysis platform (www.estimationstats.com). All of the estimation statistical results for the experiments below are found in Tables 1 and 2.

**Table 1:** Data analysis estimation statistics

Figure	Plot	Analysis	Control group	Test group	Control_N	Test_N	Effect_size	Is_paired	Difference	ci_width	ci_lower_limit	ci_upper_limit
Figure 2*B*	Multiple two groups	Analysis #1	CV/VEH	CV/CNO	7	7	Mean difference	False	–11.97857143	95.00%	–55.23714286	33.12428571
Figure 2*B*	Multiple two groups	Analysis #1	M4/VEH	M4/CNO	6	6	Mean difference	False	–41.95	95.00%	–55.33333333	–26.9
Figure 2*C*	Multiple two groups	Analysis #2	CV/VEH	CV/CNO	7	7	Mean difference	False	–2.857142857	95.00%	–19.57142857	17.71428571
Figure 2*C*	Multiple two groups	Analysis #2	M4/VEH	M4/CNO	6	6	Mean difference	False	–20.5	95.00%	–35.33333333	–12.33333333
Figure 2*D*	Multiple two groups	Analysis #3	CV/VEH	CV/CNO	6	5	Mean difference	False	–33	95.00%	–81.66666667	13.33333333
Figure 2*D*	Multiple two groups	Analysis #3	M4/VEH	M4/CNO	10	10	Mean difference	False	121.05	95.00%	31.25	188.4
Figure 3*A*	Two groups	Analysis #4	VEH	CNO	10	12	Mean difference	False	0.1358	95.00%	–11.80348333	14.73156667
Figure 3*B*	Paired	Analysis #5	VEH	CNO	7	7	Mean difference	True	–2.714285714	95.00%	–47.14285714	46.14285714
Figure 3*C*	Paired	Analysis #6	VEH	CNO	7	7	Mean difference	True	10.41428571	95.00%	–115.8285714	139.0714286
Figure 3*D*	Paired	Analysis #7	VEH	CNO	7	7	Mean difference	True	6.857142857	95.00%	2.285714286	10.57142857
Figure 3*E*	Paired	Analysis #8	VEH	CNO	6	6	Mean difference	True	1	95.00%	–2.166666667	4
Figure 3*F*	Paired	Analysis #9	VEH	CNO	6	6	Mean difference	True	0.5	95.00%	–0.666666667	2.166666667
Figure 3*G*	Multiple paired	Analysis #10	VEH/Active	VEH/Inactive	7	7	Mean difference	True	–432.2857143	95.00%	–595	–346.2857143
Figure 3*G*	Multiple paired	Analysis #10	CNO/Active	CNO/Inactive	7	7	Mean difference	True	–450.1428571	95.00%	–529.4285714	–376.8571429
Figure 3*H*	Multiple paired	Analysis #11	VEH/Active	VEH/Inactive	6	6	Mean difference	True	–46.66666667	95.00%	–56.33333333	–34.5
Figure 3*H*	Multiple paired	Analysis #11	CNO/Active	CNO/Inactive	6	6	Mean difference	True	–42.66666667	95.00%	–69.5	–21.5
Figure 3*I*	Multiple paired	Analysis #12	VEH/Active	VEH/Inactive	6	6	Mean difference	True	–16	95.00%	–25.66666667	–9.833333333
Figure 3*I*	Multiple paired	Analysis #12	CNO/Active	CNO/Inactive	6	6	Mean difference	True	–21.16666667	95.00%	–26.66666667	–15.66666667
Figure 4*B*	Two groups	Analysis #13	VEH	CNO	5	6	Mean difference	False	–71.33333333	95.00%	–84.3	–56.4
Figure 5*C*	Multiple two groups	Analysis #14	VEH/SAL	VEH/DOI	4	5	Mean difference	False	1.9391	95.00%	–11.8578	12.1919
Figure 5*C*	Multiple two groups	Analysis #14	CNO/SAL	CNO/DOI	4	5	Mean difference	False	2.87635	95.00%	–7.7447	17.15425
Figure 5*D*	Multiple two groups	Analysis #15	VEH/SAL	VEH/DOI	4	5	Mean difference	False	–11.7635	95.00%	–29.804	15.1315
Figure 5*D*	Multiple two groups	Analysis #15	CNO/SAL	CNO/DOI	6	5	Mean difference	False	35.83333333	95.00%	11.43333333	55.36666667
Figure 5*E*	Multiple two groups	Analysis #16	VEH/SAL	VEH/DOI	4	5	Mean difference	False	2.45	95.00%	–0.9	6.25
Figure 5*E*	Multiple two groups	Analysis #16	CNO/SAL	CNO/DOI	6	5	Mean difference	False	9.933333333	95.00%	–2.166666667	17.56666667
Extended Data Figure 3-1	Two groups	Analysis #17	VEH	CNO	10	12	Mean difference	False	–29.94166667	95.00%	–99.88833333	17.455
Extended Data Figure 5-1	Multiple two groups	Analysis #18	VEH/SAL	VEH/DOI	4	5	Mean difference	False	4.23	95.00%	–14.915	25.525
Extended Data Figure 5-1	Multiple two groups	Analysis #18	CNO/SAL	CNO/DOI	4	5	Mean difference	False	1.545	95.00%	–28.445	22.1

VEH, peripheral vehicle injection; CNO: peripheral CNO injection; SAL, saline microinjection through cannula; DOI, DOI microinjection through cannula; M4, hM4Di expressing mice; CV, control vector expressing mice; active, active lever presses; inactive, inactive lever presses.

**Table 2:** Data analysis estimation statistics (continued)

Figure	*p*value_permutation	*p*value_welch (unpaired) *p* value_wilcoxon (paired)	Statistic_welch (unpaired) statistics_wilcoxon (paired)	Statstics_students_t	*p*value_students_t	*p*value_mann_whitney (f or unpaired only)	Statistic_mann_whitney (for unpaired only)
Figure 2*B*	0.6066	0.629426086	0.495375657	0.629281775	0.495375657	0.522903235	30
Figure 2*B*	0.001	0.0025462	5.280732317	0.000357269	5.280732317	0.004771822	36
Figure 2*C*	0.7434	0.780895648	0.28500749	0.780497573	0.28500749	0.405717361	31.5
Figure 2*C*	<0.0001	0.019474862	3.342934417	0.00745355	3.342934417	0.004922036	36
Figure 2*D*	0.2458	0.257043461	1.216671684	0.275502716	1.161015779	0.359117043	20.5
Figure 2*D*	0.009	0.009988934	–2.907948962	0.009382872	–2.907948962	0.007936749	14.5
Figure 3*A*	0.985	0.984908385	–0.01923677	0.985863342	–0.017941451	0.575156577	69
Figure 3*B*	0.9092	0.735316691	12	0.917871783	0.107533007		
Figure 3*C*	0.9066	1	14	0.889801416	–0.144547332		
Figure 3*D*	0.0106	0.034287968	1.5	0.024737753	–2.97683363		
Figure 3*E*	0.4992	0.587936746	5.5	0.584651914	–0.583874208		
Figure 3*F*	0.4972	0.592980098	2	0.56231227	–0.620173673		
Figure 3*G*	<0.0001	0.017960478	0	0.000471094	6.863625223		
Figure 3*G*	<0.0001	0.017960478	0	4.51591E–05	10.44513808		
Figure 3*H*	<0.0001	0.027281171	0	0.000583676	7.716104886		
Figure 3*H*	0.03	0.027707849	0	0.024560107	3.179204483		
Figure 3*I*	<0.0001	0.027707849	0	0.010667223	3.967077629		
Figure 3*I*	<0.0001	0.027707849	0	0.000967699	6.918130826		
Figure 4*B*	<0.0001	7.98489E-06	9.087888669	8.98533E-06	8.943968115	0.007969413	30
Figure 5*C*	0.801	0.783198318	–0.287931746	0.798052056	–0.265799365	0.713303174	8
Figure 5*C*	0.641	0.694505354	–0.414121203	0.68071095	–0.429152553	0.90252325	9
Figure 5*D*	0.398	0.383625129	0.936849955	0.411998697	0.872230812	0.270344141	15
Figure 5*D*	0.0188	0.021465765	–2.877016972	0.016492641	–2.939779731	0.022173545	2
Figure 5*E*	0.3382	0.284355669	–1.185753812	0.314531309	–1.083399811	0.536878456	7
Figure 5*E*	0.0966	0.107754724	–1.838105924	0.090221702	–1.897682889	0.081439732	5
Extended Data Figure 3-1	0.3116	0.331880624	1.009447278	0.296991141	1.070847778	0.575156577	69
Extended Data Figure 5-1	0.7258	0.739162512	–0.348555602	0.733403483	–0.354499861	0.713303174	8
Extended Data Figure 5-1	0.9136	0.917137464	–0.108132236	0.916803225	–0.108291446	0.713303174	8

## Results

### IPN-vHipp pathway

**Figure 1.**
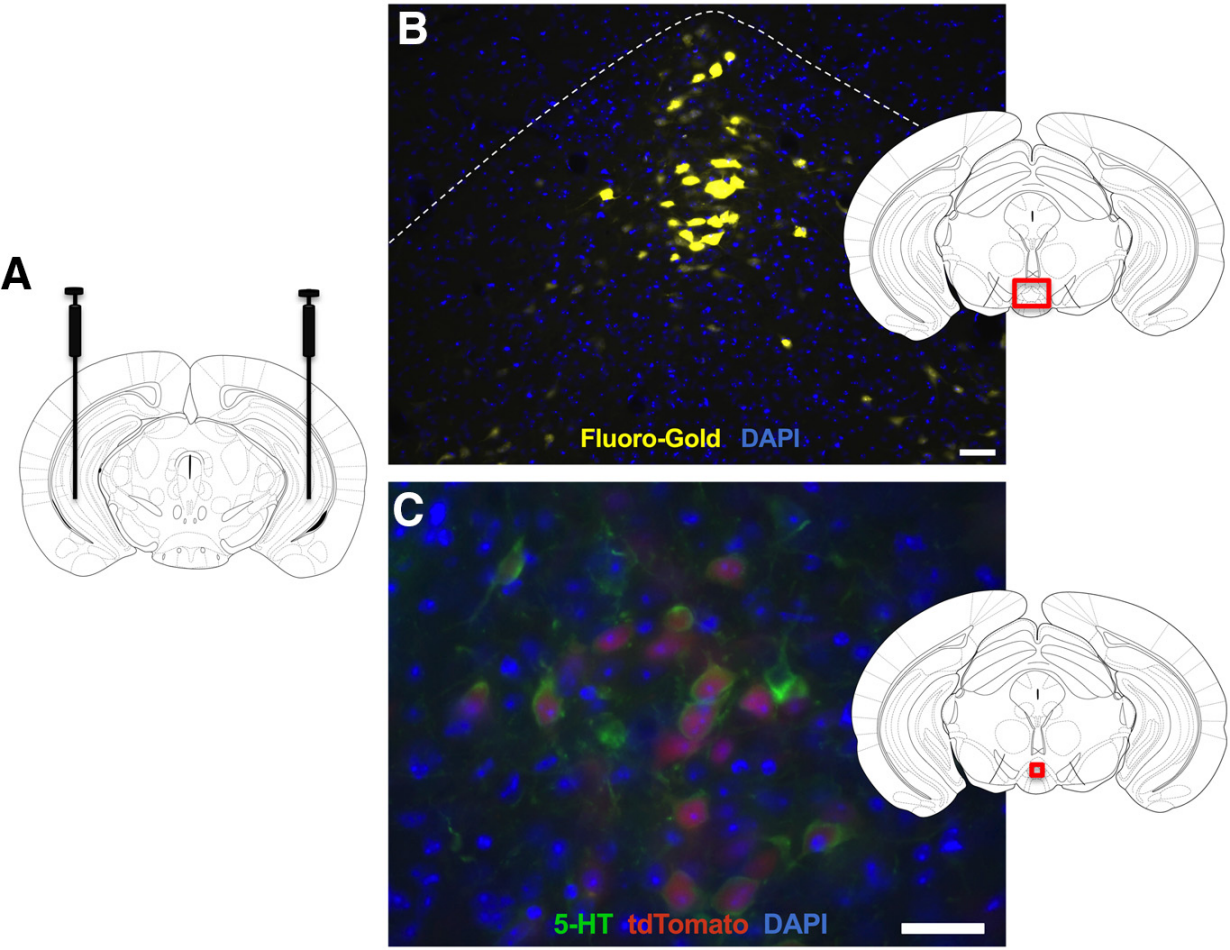
Serotonergic interpeduncular-hippocampal pathway. ***A***, Schematic displaying injections sites of the retrograde tracer and virus in the vHipp. ***B***, Lower-magnification image shows localization of cell bodies in the IPN following injection of the Fluoro-Gold (yellow) retrograde tracer bilaterally in the vHipp. Schematic indicates red bounding box for area displayed in the photomicrograph. DAPI: blue. Scale bar = 40 µm. ***C***, Serotonin (green; 5-HT) immunolabeling colocalizes with IPN-vHipp projection neurons (red; tdTomato). Rosa-tdTomato mice were injected in the vHipp with the retrograde cre-expressing AAV. Schematic indicates red bounding box for area displayed in the photomicrograph. DAPI: blue. Scale bar = 40 µm.

We first sought to validate the hippocampal coordinates containing axonal terminals from IPN-projection neurons. Based on prior studies (Wirtshafter et al., 1986), we focused on the vHipp (Fig. 1*A*). In the first experiment, wild-type mice were injected with the Fluoro-Gold retrograde tracer in the vHipp, and small clusters of neurons were found localized in the rostral IPN region, in addition to scattered cells in ventral IPN regions (Fig. 1*B*). Next, to more specifically examine whether these projection cells expressed serotonin, Rosa-tdTomato reporter mice were injected in the vHipp with the retrograde AAV Cre-expressing vector and sections were processed for immunolabeling. The prior findings were replicated, in which small clusters of neurons were visualized within the rostral IPN subregion. Further, the IPN-vHipp projection neurons were positive for serotonin immunolabeling (Fig. 1*C*), thereby confirming that this population of cells in the IPN expresses serotonin and innervates the vHipp.

### IPN-vHipp pathway regulates stress coping behaviors and natural reward consumption

**Figure 2.**
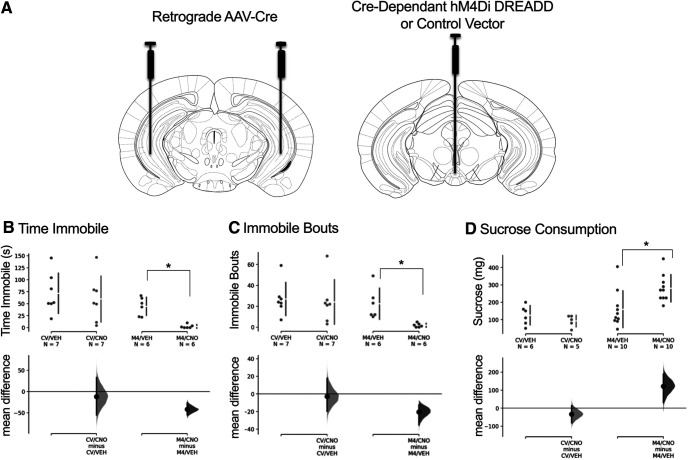
Inhibition of the IPN-vHipp pathway dramatically increases active escape behaviors and sucrose consumption. ***A***, Mice were injected with the retrograde AAV-cre bilaterally in the vHipp and the cre-dependent AAV-hM4Di DREADD (M4) or AAV control vector (CV) in the IPN. Mice expressing hM4Di in the IPN-vHipp exhibited a decrease in (***B***) time immobile and (***C***) number of immobile bouts following CNO injections relative to vehicle (VEH) injections. No differences were found in the control vector mice (CV) injected with CNO or vehicle. ***D***, CNO-mediated inhibition of hM4Di-expressing IPN-vHipp neurons also increased sucrose consumption. No differences were found in the control mice injected with CNO or vehicle; *p < 0.05.

To examine the effects of inhibiting the IPN-vHipp circuit, an intersectional chemogenetic strategy was employed to target the projections from the rostral IPN region. Mice were injected with the retrovirus expressing cre bilaterally in the vHipp and with the virus containing a floxed cre-dependent hM4Di-mCherry DREADD in the IPN (Fig. 2*A*). CNO activation of the modified human M4 muscarinic DREADD, which is coupled to G_i_ signaling, has been shown to silence neuronal activity (Armbruster et al., 2007). As a comparison, a separate group of mice were injected with the retrovirus expressing cre bilaterally in the vHipp and a control DREADD vector in the IPN. This important control condition was necessary to establish whether CNO or its metabolites would alter behavior independent of hM4Di expression (Gomez et al., 2017; Manvich et al., 2018). Mice expressing the inhibitory hM4Di or control DREADD in the IPN-vHipp pathway were then examined for behavioral differences using a stress coping behavioral assessment. An increase in time immobile and number of immobile bouts is indicative of an increase in passive coping, whereas active coping behavior is represented by an opposing behavioral response (Castagne et al., 2011; Coffey et al., 2020). We found that CNO-mediated hM4Di inhibition of the IPN-vHipp pathway resulted in a dramatic decrease in time immobile, whereas no differences were found in control mice lacking the DREADD receptor. The mean difference between M4/vehicle and M4/CNO was −41.9 [95.0%CI −55.3, −26.9], with a *p* value for the two-sided permutation *t* test at 0.001. The mean difference between control vector/vehicle and control vector/CNO was −12.0 [95.0%CI −55.2, 33.1], with a *p* value of the two-sided permutation *t* test at 0.607 (Fig. 2*B*). We also found that CNO-mediated inhibition of the IPN-vHipp resulted in a decrease in the number of immobile bouts compared with vehicle, with no differences of CNO or vehicle in control vector mice. The mean difference between M4/vehicle and M4/CNO was −20.5 [95.0%CI −35.3, −12.3], with a *p* value of the two-sided permutation *t* test at <0.0001. The mean difference between control vector/vehicle and control vector/CNO was −2.86 [95.0%CI −19.6, 17.7], with a *p* value of the two-sided permutation *t* test at 0.743 (Fig. 2*C*). To further examine the function of this pathway, sucrose consumption was used as a measure of natural reward consummatory behavior (Pushkin et al., 2019). IPN-vHipp hM4Di-expressing mice exhibited a significant increase in sucrose consumption following the CNO injection, as compared with vehicle. No behavioral differences were found in the control vector mice with CNO or vehicle injection. The mean difference between M4/vehicle and M4/CNO was 1.21e+02 [95.0%CI 31.2, 1.88e+02], with the *p* value of the two-sided permutation *t* test at 0.009. The mean difference between control vector/vehicle and control vector/CNO was −33.0 [95.0%CI −81.7, 13.3], with a *p* value of the two-sided permutation *t* test at 0.246 (Fig. 2*D*).

### Circuit inhibition alters food self-administration, not other IPN-related or vHipp-related behaviors

**Figure 3.**
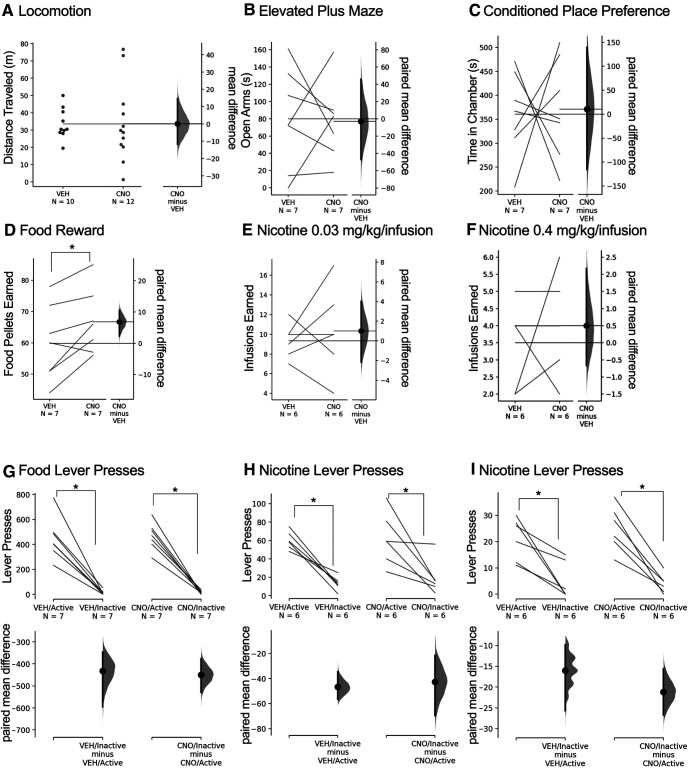
Inhibition of the IPN-vHipp pathway increases food self-administration, but not other behaviors. ***A***, Mice expressing hM4Di DREADD in the IPN-vHipp pathway did not differ in distance traveled in an open field following vehicle (VEH) or CNO injection. They also did not differ in the time in the center of the open field (see Extended Data Fig. 3-1). ***B***, Differences were also not found in anxiety-associated behavior in the EPM in IPN-vHipp hM4Di-expressing mice following VEH or CNO injections. ***C***, In the conditioned place preference task, IPN-vHipp hM4Di-expressing mice demonstrated no differences in time spent in the vehicle-paired versus CNO-paired chamber. ***D***, For food self-administration, IPN-vHipp hM4Di mice exhibited a significant increase in the number of food pellets earned following CNO injection, as compared with vehicle injection. ***E***, ***F***, When examined for intravenous nicotine self-administration, IPN-vHipp hM4Di-expressing mice did not differ in the number of nicotine infusions earned either at the (***E***) low 0.03 mg/kg/infusion or (***F***) high 0.4 mg/kg/infusion nicotine doses following vehicle or CNO injections. ***G–I***, Lever pressing behavior was examined for differences in responding between the active and inactive levers. All groups exhibited significantly higher lever pressing directed at active lever, as compared with the inactive lever, for (***G***) food reward, (***H***) 0.03 mg/kg/infusion nicotine, and (***I***) 0.4 mg/kg/infusion nicotine. Experimental design for virus injections consistent with Figure 2*A*; *p < 0.05.

Escape behaviors under stress may be mitigated by varying factors, such as generalized behavioral movements and/or anxiety-associated effects. In addition, the IPN has been implicated in aversion and nicotine self-administration (Fowler et al., 2011; Fowler and Kenny, 2014), and the vHipp in anxiety-associated behavior and learned aversion (Kenney et al., 2012; Çalışkan and Stork, 2019; Hjorth et al., 2019; Padilla-Coreano et al., 2019). Thus, we next examined mice in a series of behavioral assessments to verify the specificity of the resulting effects with DREADD-mediated inhibition of the IPN-vHipp circuit. First, locomotor activity in the open field was examined. Subjects expressing hM4Di in the IPN-vHipp were injected with CNO or vehicle, but no differences were found in the distance traveled. The mean difference between vehicle and CNO was 0.136 [95.0%CI −11.8, 14.7], with the *p* value of the two-sided permutation *t* test at 0.985 (Fig. 3*A*). Differences were also not found in the time spent in the center of the open field. The mean difference between vehicle and CNO was −29.9 [95.0%CI −99.9, 17.5], with the *p* value of the two-sided permutation *t* test at 0.312 (Extended Data Fig. 3-1). Mice also did not differ in the time spent in the open arms of the EPM. The mean difference between vehicle and CNO was −2.71 [95.0%CI −47.1, 46.1], with the *p* value of the two-sided permutation *t* test at 0.909 (Fig. 3*B*). Next, to examine if inhibition of this circuit induces an aversive or rewarding effect, mice were tested in the conditioned place preference procedure, in which each chamber was paired with either a vehicle or CNO injection during conditioning sessions. IPN-vHipp hM4Di mice demonstrated no significant difference in the time spent in either paired chamber, indicating that inhibition of this pathway does not independently induce aversion or reward effects. The mean difference between vehicle and CNO was 10.4 [95.0%CI −1.16e+02, 1.39e+02], with the *p* value of the two-sided permutation *t* test at 0.907 (Fig. 3*C*). Self-administration behaviors were next examined to assess reward and aversion under an effortful fixed ratio 5, time out 20-s schedule of reinforcement. First, hM4Di IPN-vHipp mice were examined for lever pressing behavior to earn food pellets. A significant increase in the number of food rewards earned was found following CNO injection, compared with vehicle control. The mean difference between vehicle and CNO was 6.86 [95.0%CI 2.29, 10.6], with the *p* value of the two-sided permutation *t* test at 0.0106 (Fig. 3*D*). This level of food self-administration for the control was consistent with prior studies (Fowler and Kenny, 2011; Chen et al., 2018). Next, mice were examined for intravenous nicotine self-administration at the lower acquisition dose of nicotine (0.03 mg/kg/infusion; Fowler and Kenny, 2011). No significant difference was found for the number of infusions earned following CNO or vehicle administration at the 0.03 mg/kg/infusion nicotine dose. The mean difference between vehicle and CNO was 1.0 [95.0%CI −2.17, 4.0], with the *p* value of the two-sided permutation *t* test at 0.499 (Fig. 3*E*). Finally, given that the IPN has been selectively implicated in regulating intake at higher doses of nicotine (Fowler et al., 2013), we examined responding at the high 0.4 mg/kg/infusion dose, but no significant differences were found following CNO or vehicle injection. The mean difference between vehicle and CNO was 0.5 [95.0%CI −0.667, 2.17], with the *p* value of the two-sided permutation *t* test at 0.497 (Fig. 3*F*). Both vehicle and CNO groups exhibited a significant increase in their active lever presses versus inactive lever presses during food training and nicotine self-administration, demonstrating a specific association with the active lever for food or nicotine reward, respectively. For lever pressing during food training, the mean difference between vehicle/active and vehicle/inactive was −4.32e+02 [95.0%CI −5.95e+02, −3.46e+02], with the *p* value of the two-sided permutation *t* test at <0.0001. The mean difference between CNO/active and CNO/inactive was −4.5e+02 [95.0%CI −5.29e+02, −3.77e+02], with the *p* value of the two-sided permutation *t* test at <0.0001 (Fig. 3*G*). For lever pressing with the 0.03 mg/kg/infusion nicotine self-administration sessions, the mean difference between vehicle/active and vehicle/inactive was −46.7 [95.0%CI −56.3, −34.5], with the *p* value of the two-sided permutation *t* test at <0.0001. The mean difference between CNO/active and CNO/inactive was −42.7 [95.0%CI −69.5, −21.5], with the *p* value of the two-sided permutation *t* test at 0.03 (Fig. 3*H*). For lever pressing behavior with the 0.4 mg/kg/infusion nicotine self-administration sessions, the mean difference between vehicle/active and vehicle/inactive was −16.0 [95.0%CI −25.7, −9.83], with the *p* value of the two-sided permutation *t* test at <0.0001; the mean difference between CNO/active and CNO/inactive was −21.2 [95.0%CI −26.7, −15.7], with the *p* value of the two-sided permutation *t* test at <0.0001 (Fig. 3*I*).

10.1523/ENEURO.0191-20.2020.f3-1 Extended Data Figure 3-1 Mice expressing hM4Di DREADD in the IPN-vHipp pathway did not differ in time spent in the center of an open field following vehicle (VEH) or CNO injection. Download Figure 3-1, TIF file.

### DREADD-mediated inhibition decreases IPN-vHipp neuronal activation

**Figure 4.**
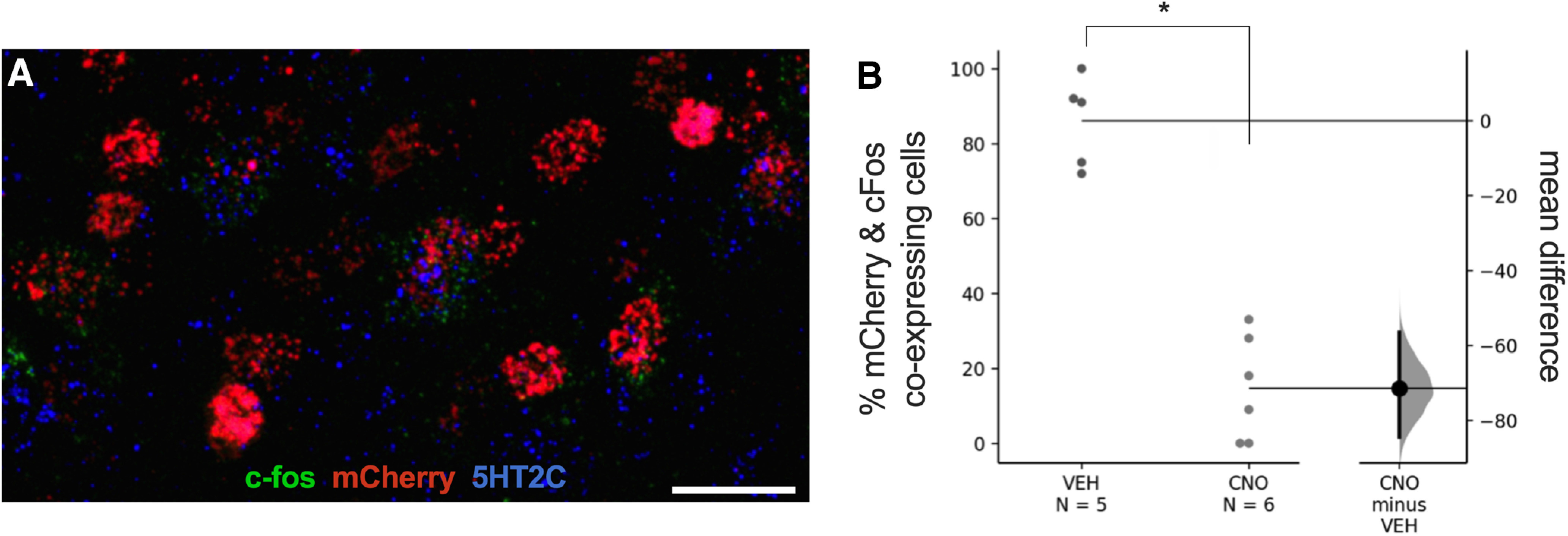
Differences in IPN-vHipp pathway neuronal activation in mice expressing the inhibitory hM4Di DREADD. ***A***, C-fos (green) was expressed in hM4Di IPN-vHipp neurons (red, mCherry) that co-expressed the serotonin receptor 5-HT_2C_ (blue) as revealed with RNAscope. Scale bar = 10 µm. ***B***, Quantification of c-fos protein expression in IPN-vHipp hM4Di mice injected with vehicle (VEH) or CNO before the stress coping behavioral assessment. CNO treatment induced a statistically significant reduction in the percentage of mCherry-positive cells co-expressing c-fos, thus validating hM4Di-induced inhibition of this cell population. Experimental design for virus injections consistent with Figure 2*A*; *p < 0.05.

To further validate the effects of CNO-induced hM4Di-mediated inhibition of the IPN-vHipp, brain tissue was examined from mice following the stress coping behavioral assessment. First, RNAScope analysis was conducted with probes targeting cfos, mCherry, and the serotonin 2C receptor 5-HT_2C_. Colocalization of c-fos, mCherry, and 5-HT_2C_ was evidenced in the IPN (Fig. 4*A*). Thus, brain sections were next processed for mCherry and c-fos immunoreactivity to quantify the number of IPN-vHipp cells expressing both of these markers; the total number of mCherry-positive cells quantified for the vehicle-injected and CNO-injected groups was 54 and 50, respectively. IPN-vHipp hM4Di mice injected with CNO demonstrated a significant decrease in the percentage of mCherry virus expressing cells that co-localized with c-fos, as compared with vehicle injection. The mean difference between vehicle and CNO was −71.3 [95.0%CI −84.3, −56.4], with the *p* value of the two-sided permutation *t* test at <0.0001 (Fig. 4*B*).

### Serotonergic signaling underlies IPN-vHipp behavioral effects

**Figure 5.**
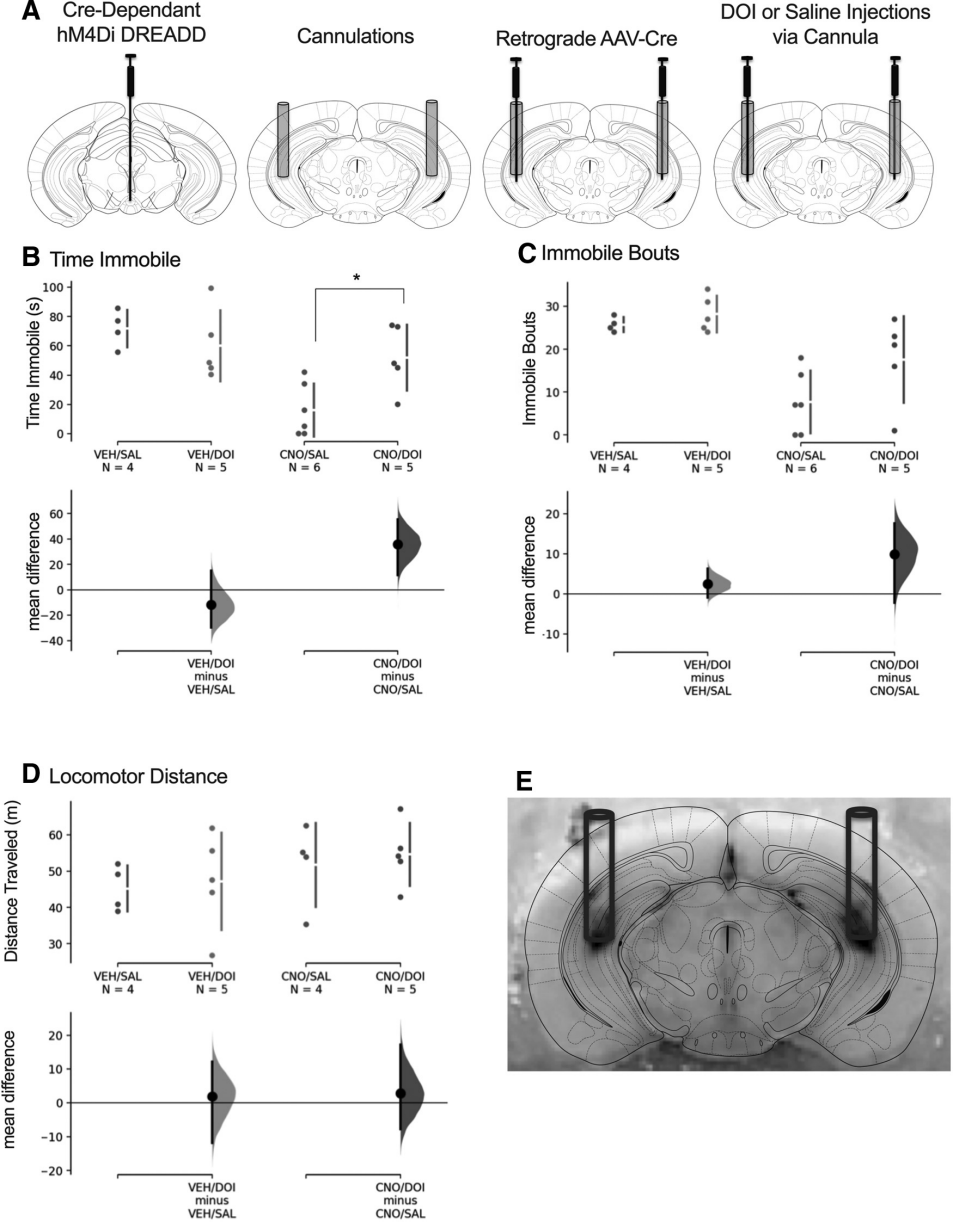
Site-specific injection of a 5-HT_2A/2C_ agonist (DOI) reverses CNO-induced active escape behavior in IPN-vHipp hM4Di-expressing mice. ***A***, Schematic illustrates location of injections and cannula. Mice were first injected with a cre-dependent hM4Di DREADD AAV into the IPN (left) and were then cannulated bilaterally in the vHipp (center left). Retrograde AAV-cre injections were administered 1 mm below the cannula tip (center right). Following subcutaneous injections of CNO or vehicle (VEH), freely moving mice were then microinjected with DOI or saline (SAL) in the vHipp via the guide cannula (right) before the behavioral task. ***B–D***, Mice treated with both CNO and DOI exhibited an increase in time immobile (***B***) and a trend of an increase in the number of immobile bouts (***C***), demonstrating that activating 5-HT2A/2C receptors reverses the effects of IPN-vHipp inhibition. ***D***, When examined in the open field, mice exhibited no differences across treatments in the locomotor distance traveled. They also did not differ in the time in the center of the open field (see Extended Data Fig. 5-1). ***E***, Representative brain image illustrates bilateral cannula tracks with black dye injection into the vHipp; *p < 0.05.

To further establish the serotonergic mechanisms involved in IPN-vHipp function, mice were injected with the cre-dependent hM4Di in the IPN. Thereafter, they were implanted with bilateral cannula directed into the vHipp, through which the retrograde AAV-cre virus was then injected (Fig. 5*A*,*E*). Given that the ventral pole of the hippocampus contains a high density of 5-HT_2A_ and 5-HT_2C_ receptors (Tanaka et al., 2012), our pharmacological approach was directed at these receptor subtypes. Before the stress coping assessment, mice were first injected with CNO or vehicle subcutaneously, and then were microinjected with DOI, a 5-HT_2A/2C_ agonist, or saline via the guide cannula in the vHipp. Given that the IPN-vHipp circuit was found to contain serotonergic neurons (Fig. 1*B*), we hypothesized that inhibition of the IPN-vHipp circuit would result in a decrease in serotonin release in the vHipp, leading to the increased active escape behaviors found in these studies (Fig. 2*B*,*C*). Thus, we proposed that injection of an agonist during circuit inhibition may thereby reverse the effects on serotonergic signaling, leading to a rescue of the synaptic effects of circuit inhibition on escape behaviors. DOI microinjections in the vHipp reversed the effects of CNO-mediated hM4Di DREADD IPN-vHipp inhibition on time immobile, whereas no differences were found in the vehicle-injected mice. The mean difference between CNO/saline and CNO/DOI was 35.8 [95.0%CI 11.4, 55.4], with the *p* value of the two-sided permutation *t* test at 0.0188, and the mean difference between vehicle/saline and vehicle/DOI is −11.8 [95.0%CI −29.8, 15.1], with the *p* value of the two-sided permutation *t* test at 0.398 (Fig. 5*B*). We also found a trend in DOI reversing the effects of CNO-mediated DREADD inhibition on the number of immobile bouts. The mean difference between CNO/saline and CNO/DOI was 9.93 [95.0%CI −2.17, 17.6], with the *p* value of the two-sided permutation *t* test at 0.0966. The mean difference between vehicle/saline and vehicle/DOI was 2.45 [95.0%CI −0.9, 6.25], with the *p* value of the two-sided permutation *t* test at 0.338 (Fig. 5*C*). Finally, we examined whether DOI would affect general locomotion to ensure that the effects in the above measures were not because of changes in generalized behavior, but we found no significant difference with DOI vHipp injections in the open field. The mean difference between vehicle/saline and vehicle/DOI was 1.94 [95.0%CI −11.9, 12.2], with the *p* value of the two-sided permutation *t* test at 0.801. The mean difference between CNO/saline and CNO/DOI was 2.88 [95.0%CI −7.74, 17.2], with the *p* value of the two-sided permutation *t* test at 0.641 (Fig. 5*D*). We also found no differences in the center time between mice injected with DOI and saline. The mean difference between vehicle/saline and vehicle/DOI was 4.23 [95.0%CI −14.9, 25.5], with the *p* value of the two-sided permutation *t* test at 0.726. The mean difference between CNO/saline and CNO/DOI was 1.55 [95.0%CI −28.4, 22.1], with the *p* value of the two-sided permutation *t* test at 0.914 (Extended Data Fig. 5-1).

10.1523/ENEURO.0191-20.2020.f5-1 Extended Data Figure 5-1 Mice expressing hM4Di in the IPN-vHipp were injected with CNO or vehicle (VEH) subcutaneously, and then microinjected with DOI or Saline (SAL) directly into the vHipp via dual guide cannula. No differences were found in the time spent in the center of the open field among groups following treatment. Download Figure 5-1, TIF file.

## Discussion

These studies define the function of the IPN-vHipp pathway using an intersectional chemogenetic viral approach. DREADD-mediated inhibition of the IPN-vHipp pathway substantially increased active escape behaviors, and food self-administration and natural reward consumption of sucrose. These effects were independent of any changes in motor activity or anxiety-associated behavior. Further, differences were not found in intravenous nicotine self-administration at either a low or high nicotine dose. Microinjection of a 5-HT_2A/2C_ receptor agonist in the vHipp during IPN-vHipp circuit inhibition was found to reverse active escape efforts, without any differences in locomotion. These data provide further evidence that serotonergic signaling from the IPN-vHipp pathway mediates coping behavior.

### Role of the IPN-vHipp in stress coping behavior

In humans, disrupted hippocampal homeostasis has been linked to altered stress coping (Posener et al., 2003), and increased hippocampal activity is found in patients suffering from major depression (Milne et al., 2012). During inhibition of the IPN-vHipp pathway, we found that mice exhibited a dramatic increase in escape attempts within the water chamber, resulting in minimal time immobile. In this assessment, mice may either increase or decrease their swimming behavior; increased swimming behavior is thought to reflect an increase in active escape responses, whereas a decrease in swimming behavior is associated with a more passive coping profile (Commons et al., 2017; Coffey et al., 2020). However, excessive responses at both ends of this spectrum can be indicative of maladaptive behavior. For instance, excessive passive coping responses can reflect an anhedonia-associated state (e.g., behavioral expression of helplessness). In contrast, excessive active escape responses may be characteristic of a state of panic and/or mania-associated behavior, in which an animal struggles to escape despite increasing exhaustion that could result in a more severe health outcome (e.g., inability to perform minimal behaviors to support passive coping strategies). Of note, in the present study, we single housed mice to induce a state of mild stress (Bächli et al., 2008), with the goal of being able to assess a relative increase or decrease in passive stress coping behavior (Porsolt, 2000; Manouze et al., 2019). Thus, the extreme behavioral profile exhibited by the mice with inhibition of the IPN-vHipp and under such stress conditions may be indicative of maladaptive, excessive active escape responses. This effect was specific to stress coping behavior, as no differences were found in locomotor or anxiety-related behaviors.

Interestingly, it has been well established that globally increasing serotonin levels in the synaptic region increases active coping behavior, for instance with administration of SSRIs (Cryan et al., 2005; Mezadri et al., 2011). Further, acute activation of serotonergic neurons in the dorsal raphe nucleus similarly increases active coping behavior, while inhibition of the raphe neurons increases anxiety-related behaviors (McDevitt et al., 2011; Nishitani et al., 2019). Importantly, in our studies, we found an opposing effect on serotonergic modulation, in which inhibition of the serotonergic IPN-vHipp pathway increased active escape behaviors. Further, this DREADD-mediated inhibitory effect was reversed by administration the 5-HT_2A/2C_ agonist, DOI, in the vHipp. This region contains a high density of 5-HT_2A_ and 5-HT_2C_ receptors (Tanaka et al., 2012), both of which may be expressed on the presynaptic or postsynaptic terminal. Thus, further studies will be necessary to delineate the specific receptor localization that mediates these behavioral effects within the vHipp. Taken together, serotonin signaling appears to exhibit opposing effects in a circuit-specific manner, which may have important implications for global serotonergic manipulation with therapeutic SSRI approaches.

### Role of the IPN-vHipp in reward-related feeding behaviors

We also found that DREADD-mediated inhibition of the IPN-vHipp pathway increased natural reward consumption for sucrose and food reinforcement. This is consistent with prior studies demonstrating that 5HT_2A_ and 5HT_2C_ agonists decrease food pellets earned during food training (De Vry et al., 2003; Howell et al., 2019). Blunted sucrose intake has been proposed to reflect impaired sensitivity to reward as a model of anhedonia (Monleon et al., 1995). Interestingly, inhibition of the IPN-vHipp circuit was not rewarding by itself, as evidenced in the conditioned place preference test. Further, inhibition of the pathway also did not alter the rewarding or aversive value of nicotine, at either a low or high dose. Taken together, these findings support the notion that inhibition of the IPN-vHipp circuit may enhance the incentive value of natural rewards. It is further possible that this response may be related to a potential mania-associated state, which would be consistent with the excessive escape behaviors discussed above. However, this possibility needs to be further examined with additional behavioral models of mania (Young et al., 2011).

### Considerations based on the experimental approach

It has been proposed that CNO may back-metabolize into clozapine and exert effects on cellular signaling (Manvich et al., 2018). Therefore, we selected a relatively low dose of CNO in these studies (Marchant et al., 2016; Mahler et al., 2019) and employed important control conditions to allow for proper interpretation of the findings. Of note, we found no effects of CNO alone in our control groups on the behavioral assessments. Further, the relative amount of back metabolized clozapine occurring within the vHipp following a subcutaneous injection at the dose provided is unknown. Thus, the peripheral injection of CNO did not appear to produce interoceptive stimulus effects via the vHipp in these studies, as evidenced with the control conditions. However, it would be interesting in future studies to inject clozapine into the vHipp to specifically determine if any behavioral differences can be induced. It is also interesting to note that DOI has been shown to elicit hallucinogenic properties in humans (Aghajanian and Marek, 1999). It is unknown as to whether DOI in the vHipp specifically produces these hallucinogenic effects, and it is additionally extremely difficult to assess the presence of hallucinations in mice. However, this factor may have played a role in the behaviors exhibited. It will also be of interest in future studies to assess the specific cellular effect of DOI in reversing DREADD-induced inhibition. Interestingly, an *in vitro* study found that DOI applied with a high frequency pulse train induced plasticity changes within 30 min in the amygdala (Chen et al., 2003). Although structural plasticity has been noted ∼24 h after DOI exposure *in vivo* (Ly et al., 2018), it is still possible that receptor activation in the vHipp by either DOI or endogenous serotonergic signaling (e.g., IPN-vHipp activation in the absence of hM4Di inhibition) could have resulted in changes in synaptic plasticity. Next, mice were socially isolated after AAV injections for three weeks before the assessment of stress coping behavior, and thus, it is possible that mice in a reduced or increased stress state may exhibit differential effects with inhibition of the IPN-vHipp pathway. Finally, we examined DREADD mediated inhibition of the IPN-vHipp pathway. It will be of further interest in future studies to determine if microinjecting a 5-HT_2A/2C_ antagonist into the vHipp would override the effects of activating the IPN-vHipp pathway, such as with hM3Dq DREADD expression.

### Potential translational relevance to human symptomology

A maladaptive response to stressful situations is characteristic of many psychiatric disorders and likely involves imbalance in various neurotransmitter systems, such as serotonin (Must et al., 2007) and dopamine (Meyer et al., 2001). Indeed, patients at risk of stress coping maladaptive responses, such as that found in depression, are found to have persistent abnormalities in brain serotonin mechanisms (Deakin et al., 1990; Cowen, 2008). Different brain regions have been implicated in stress coping dysfunction, including the hippocampus (Fujita et al., 2000) and raphe nuclei (Lira et al., 2003). Lowering brain serotonin activity through tryptophan deletion in recovered patients produces acute symptomatic relapse (Cowen, 2008). However, increasing global serotonin levels with SSRIs is not an efficacious treatment for all individuals diagnosed with depression (Nestler et al., 2002; Blier, 2009). Our findings suggest that this discrepancy may be attributed to opposing serotonin pathways and function.

### Conclusions

In these studies, we discovered that the novel serotonergic IPN-vHipp pathway modulates stress coping responses and natural reward. Of importance, these findings challenge the canonical understanding of serotonin by demonstrating that inhibition of serotonergic signaling selectively in the IPN-vHipp results in similar behavioral effects as that found with increased global brain serotonin (e.g., with SSRI treatment in the behavioral assessments). These findings highlight opposing serotonin-mediated effects in a brain circuit specific manner. It will be important in future studies to further discern whether the vHipp acts as a signal integration center or if downstream pathways further propagate this signaling to affect broader circuit function. Finally, consideration of the opposing serotonergic pathways may also lead to novel approaches to treat symptomology associated with psychiatric disorders.

## Synthesis

Reviewing Editor: Nathalie Ginovart, University of Geneva Decisions are customarily a result of the Reviewing Editor and the peer reviewers coming together and discussing their recommendations until a consensus is reached. When revisions are invited, a fact-based synthesis statement explaining their decision and outlining what is needed to prepare a revision will be listed below. The following reviewer(s) agreed to reveal their identity: Michael Scofield, Vidita Vaidya.

Upon discussion with the two reviewers, we all agreed that the manuscript is interesting and informative but several concerns have been raised that should be addressed in the revision. In particular, a major concern relates to the disparity in the effect of IPN-vHipp inhibition on stress coping when comparing Fig. 2a-b and Fig. 5b-c. that may hinder interpretation of the results. There were also concerns about the statistical analyses which stems in part from the potential presence of outliers in the data. Normality of data distribution and homogeneity of variance between groups need to be checked and appropriate statistical tests used accordingly. Moreover, the statistical approach used to test the effect of IPN-vHipp inhibition in the stress coping behavioral assessment and sucrose consumption was deemed not optimal and both reviewers recommended using a two-way ANOVA for comparison of CNO effect with the control vector group. Both reviewers also had suggestions during online discussion for adding a Vehicle+DOI group for comparison with the CNO+DOI group in Fig. 5 to exclude a DOI effect per se on active escape behaviors and assure robustness of the conclusion. We realize collecting more data may be difficult, but the authors should either collect those data or acknowledge this shortcoming in the revision and discuss it. There were several minor comments and requests for methodological clarifications from both reviewers that should be addressed.

Please refer to specific reviewers’ comments below. In addition to their comments, please also address my comments below:

1) There are sometimes major discrepancies between the numbers of animals reported to be used for each group in the methods and the number of animal actually presented in the results and figures. For instance in experiment 1 or in the control vector experiment, a total of 12 animals per experiment (n=6 per group I guess) were used but the data of only 4 or 5 per group are displayed in Fig. 2 (and even 7 animals /group for the sucrose test). The degrees of freedom reported for t-tests in the results are also erroneous. This should be corrected. Moreover, the authors should clarify the reason why some data points are missing in some figure panels, especially in Fig 2 whose data are central to the paper.

2) Regarding the lack of clarity in the methodological detail provided, it is not clear in Fig. 5 whether mice were first injected with CNO or vehicle and then microinjected with DOI or vehicle, as stated in the methods (line 178-180), or whether they were first injected with CNO and then microinjected with DOI, as stated in the results (line 445) and legends of Fig. 5. Please revise.

Reviewer 1

The authors aimed to gain an understanding of a previously unidentified circuit (IPN-vHipp) in maladaptive coping with stress. The authors establish a role for this circuit in stress-induced coping behaviors, as well as general reward consumption. Importantly, the authors show that the effect of inhibition of the pathway can be reversed by engaging 5-HT2A/2C receptors in the vHipp, although this interpretation is slightly hindered by a lack of indication that the authors replicated the results shown in figure 2 when performing the experiment shown in figure 5. Overall, the methods are generally sound, and the results are informative. I have several minor concerns, two major concerns, and recommendations for improving the manuscript visually.

Major concerns:

1. For cannulation experiments, it would be helpful to show a map of the ventral tip of all cannula placements. On this note, figure 5 should include a schematic to more clearly illustrate that these animals expressed DREADD specifically in the IPN-vHipp pathway and that CNO was administered. This was not abundantly clear in the methods and was only clear in the results and figure legend. Furthermore, if I understand the authors correctly, all animals in this experiment expressed DREADDs in the IPN-vHipp pathway and all received CNO, yet the data in the vehicle microinfusion group are quite different than the CNO group in Figure 2. Did the CNO manipulation actually work in Figure 5? If not, it is questionable whether the authors can conclude that their pharmacological approach reversed the effect of DREADD inhibition of the pathway. A statistical comparison between the CNO injected animals in Figure 2 and the CNO + Vehicle microinfusion group in Figure 5 would be needed (perhaps a supplement).

2. It would be appropriate to analyze figure 2a and 2d together, 2b and 2e, as well as 2c and 2f together using a two-way ANOVA. these aren’t really separate experiments. this is especially relevant given that it appears there may be an effect of virus (i.e. vehicle in 2a is lower than vehicle in 2d, etc). On this note, were there any tests for outliers performed? if not, why? it appears that there are outliers in 2d and 2e. Moreover, were data tested for assumptions of normality? What about differences in variance between groups?

Minor concerns:

1. There are a few typos in the significance statement as well as scattered throughout the manuscript; there are additional grammatical issues.

2. the authors show effects on sucrose consumption. It would be helpful to understand why the authors chose to not conduct a sucrose preference test as this is likely more valid for understand stress-induced anhedonia.

3. The authors should include a citation/justify their use of the CNO dose. Moreover, this dose of CNO is more than sufficient to be back metabolized to clozapine and produce clozapine-like interoceptive stimulus effects in mice (Manvich et al., 2018). Given that clozapine has antagonist effects at 5-HT 2A/2C receptors, this needs to be discussed. Also why was subcutaneous administration of CNO selected?

4. An experimental design flowchart would be helpful.

5. A figure with nicotine and food self-administration across sessions would be ideal, including active, inactive, and infusions earned. Were lever presses different between groups? Were CS cue presentations paired with infusions? Also, motivation to seek nicotine is typically carried out with extinction and then reinstatement. Given the authors did not do this, this should be discussed as a limitation. What the authors are measuring is an index of nicotine reward, not motivation.

6. DOI also has actions at serotonin 2B receptors. Moreover, DOI is known to have hallucinogenic properties. Does DOI microinfused into the vHipp induce a hallucinogenic state? Discuss. On that note, the authors should discuss what the cellular effect of DOI in reversing DREADD-induced inhibition of the IPN-vHipp may be. For example, DOI has been shown to alter structural plasticity. Discuss.

7. In line 369, the authors refer to the DREADD construct as anterograde. This is confusing, as it makes it sound like a tracer. Reword.

8. The time spent in the center of the open field (related to figure 3) should be included as a supplement given that the data was analyzed. Alternatively, provide the mean +/-SEM in the text as opposed to a statistical analysis.

9. The authors indicate on line 419 that their effect on food consumption is consistent with previous research. Cite that research, please.

10. figure 4 - some indication of the number of mcherry+ cells analyzed should be included to give the reader a better idea of what the denominator was and whether this was different between groups. Also, did the CNO manipulation decrease cfos in non-dreadd expressing neurons?

Reviewer 2

The authors dissect out a serotonergic pathway from the interpeduncular nucleus to the ventral hippocampus using intersectional genetic strategies and chemogenetic approaches to demonastrate that this pathway plays an important role in active coping, escape behaviors, as well as influencing sucrose consumption and motivation for food-related rewards. They illustrate that these effects of modulation of the IPN-vHIppocampal 5-HT pathway can be overridden by the administration of the 5-HT2A/c receptor agonist DOI via cannulae directly into the ventral hippocampus. The studies are of interest to the field and suggest a rather different role for this 5-HT pathway, contrasting from the existent literature. However, there are several points that do need to be addressed by the authors.

Specific points:

1. The authors may want to illustrate the precise genetic strategy and mode of administration of either the DREADD ligand or the 5-HT2A/C agonist above each figure in an illustrated schematic to make it easier for the reader.

2. The study was carried out in singly housed mice and that opens up the question as to whether the same effects would be noted in group housed animals that do not have an overlay of isolation stress.

3. Does activation of the IPN-vHippocampal 5-HT pathway modulate escape behaviors, either reducing active coping and toggling to passive coping strategies or resulting in reduction overall in escape behaviors?

4. Would delivering a 5-HT2A or 2C antagonost into the ventral hippocampus override the effects of activation of the serotonergic IPN-vhippocampal pathway?

5.Does delivering DOI into the ventral hippocampus per se influence overall locomotion?

## Author Response

We thank the Editors and Reviewers for their thoughtful and comprehensive analysis of the

manuscript and constructive comments. We appreciate that the reviewers and editors felt the manuscript was interesting and informative. In response to all of the comments, we have added additional experimental control studies/animals and modified the analysis of the data using estimation statistics. We believe these changes have significantly strengthened the manuscript and our experimental conclusions. Specific comments have been addressed as follows:

Significance Statement Comments:

There are a few typos in the significance statement that should be corrected. Moreover, while inhibition of the IPN-vHipp pathway indeed dramatically increased active stress-induced escape behaviors, the effects on sucrose consumption and motivation for food reward were much more modest. The corresponding sentence in the significance statement (as well as in the abstract) should be rephrased to tone down the impact of IPN-vHipp pathway inhibition on those two latter variables.

Response: The Significance Statement has been modified accordingly. We have also now

analyzed our data with estimation statistics, based on the request of Dr. Christophe Barnard, Senior Editor-in-Chief at eNeuro. Thus, the statement and following manuscript now reflects these changes. Discussion of the effect on sucrose consumption and food self-administration has also been toned down.

Synthesis Statement:

Upon discussion with the two reviewers, we all agreed that the manuscript is interesting and

informative but several concerns have been raised that should be addressed in the revision. In particular, a major concern relates to the disparity in the effect of IPN-vHipp inhibition on stress coping when comparing Fig. 2a-b and Fig. 5b-c. that may hinder interpretation of the results. There were also concerns about the statistical analyses which stems in part from the potential presence of outliers in the data. Normality of data distribution and homogeneity of variance between groups need to be checked and appropriate statistical tests used accordingly. Moreover, the statistical approach used to test the effect of IPN-vHipp inhibition in the stress coping behavioral assessment and sucrose consumption was deemed not optimal and both reviewers recommended using a two-way ANOVA for comparison of CNO effect with the control vector group.

Response: In response to these concerns and at the recommendation of the Senior Editor- in-Chief, we have now analyzed all of our data with estimation statistics. Furthermore, we have combined groups with the analysis as suggested.

Both reviewers also had suggestions during online discussion for adding a Vehicle+DOI group

for comparison with the CNO+DOI group in Fig. 5 to exclude a DOI effect per se on active escape behaviors and assure robustness of the conclusion. We realize collecting more data may be difficult, but the authors should either collect those data or acknowledge this shortcoming in the revision and discuss it.

Response: We have now added an additional control group as suggested by the reviewers and editor in the consultation summary, and these data are located in Figure 5. Importantly, we found no difference in the effects of DOI itself on active escape behaviors. In addition to the Vehicle+DOI group, we also added a Vehicle+Saline group to provide further control for these conditions and to strengthen our data interpretation. We believe that these additional control experiments have provided assurance of the robustness of the conclusion.

There are sometimes major discrepancies between the numbers of animals reported to be used for each group in the methods and the number of animal actually presented in the

results and figures...Moreover, the authors should clarify the reason why some data points are

missing in some figure panels, especially in Fig 2 whose data are central to the paper.

Response: We thank the reviewer for bringing this important point to our attention. The total animal numbers listed in the methods of the first version of the manuscript did not reflect all of the mice used in these studies, whereas the number of mice presented as data points in the graphs and statistical analyses were correct; we apologize for this initial oversight in the manuscript preparation. Specifically, another cohort of mice were used for the locomotor studies, as we sought to confirm that the repeated CNO injections did not underlie the absence of an effect with locomotion. Moreover, after the initial cohort assessment with four mice in the stress coping assessment, they were sacrificed to verify the accurate expression of the virus; in the subsequent cohorts, the mice were then further analyzed in the sucrose and locomotor conditions. In accordance with the synthesis statement, we have now also re-organized Figure 2 to incorporate the recommended two- group analysis with estimation statistics. Finally, additional mice have been added to provide further controls, and their data support the initial interpretations.

Regarding the lack of clarity in the methodological detail provided, it is not clear in Fig. 5 whether mice were first injected with CNO or vehicle and then microinjected with DOI or vehicle, as stated in the methods (line 178-180), or whether they were first injected with CNO and then microinjected with DOI, as stated in the results (line 445) and legends of Fig. 5. Please revise.

Response: We appreciate the reviewer’s suggestion to improve the clarity of our methods, and we have revised the manuscript text accordingly. All cannulated mice were first injected peripherally with CNO or vehicle, and then DOI or vehicle was injected. This order of injections was performed since we sought to counter-act the potential effect of CNO- mediated inhibition at the serotonergic axonal terminals in the hippocampus.

Reviewer 1

The authors aimed to gain an understanding of a previously unidentified circuit (IPN-vHipp) in maladaptive coping with stress. The authors establish a role for this circuit in stress-induced coping behaviors, as well as general reward consumption. Importantly, the authors show that the effect of inhibition of the pathway can be reversed by engaging 5-HT2A/2C receptors in the vHipp, although this interpretation is slightly hindered by a lack of indication that the authors replicated the results shown in figure 2 when performing the experiment shown in figure 5.

Overall, the methods are generally sound, and the results are informative. I have several minor concerns, two major concerns, and recommendations for improving the manuscript visually.

Response: We greatly appreciate the reviewer’s positive and constructive feedback. Since the mice in Figure 5 are cannulated, their behavior may differ from un-cannulated mice, which prohibited our direct statistical comparison. However, in response to this concern, we have now incorporated additional behavioral analyses for control conditions to allow for the comparison of experimental conditions within mice that have all been cannulated (Figure 5). As evidenced, CNO induced a decrease in time immobile in hM4Di-expressing mice, which replicates the findings shown in Figure 2.

Major concerns:

1. For cannulation experiments, it would be helpful to show a map of the ventral tip of all cannula placements. On this note, figure 5 should include a schematic to more clearly illustrate that these animals expressed DREADD specifically in the IPN-vHipp pathway and that CNO was administered. This was not abundantly clear in the methods and was only clear in the results and figure legend. Furthermore, if I understand the authors correctly, all animals in this experiment expressed DREADDs in the IPN-vHipp pathway and all received CNO, yet the data in the vehicle microinfusion group are quite different than the CNO group in Figure 2. Did the CNO manipulation actually work in Figure 5? If not, it is questionable whether the authors can conclude that their pharmacological approach reversed the effect of DREADD inhibition of the pathway. A statistical comparison between the CNO injected animals in Figure 2 and the CNO

+ Vehicle microinfusion group in Figure 5 would be needed (perhaps a supplement).

Response: We have now included schematics to provide clarification for the injections received by the mice. After obtaining the representative image shown in Figure 5e, cannula placement was visually verified by cross-referencing to this image and the brain atlas coordinates during tissue sectioning. All mice were verified as having the cannula correctly placed. Thereafter, the IPN sections were retained for immunohistochemical verification of viral expression. Therefore, it is currently not possible to provide a schematic map for each of the injector tip locations, as these sections were not able to be retained. As noted above, since the mice in Figure 5 are cannulated, their behavior may differ from an un-cannulated mice during the stress coping assessment since the intracranial injections into the brain immediately prior to the test may induces some stress and the cannula adds additional weight on their skulls during the swimming test. This prohibited our direct statistical comparison to the data in Figure 2. However, in response to this concern, we have now incorporated additional behavioral analyses for control conditions to allow for the comparison of experimental conditions within mice that have all been cannulated (Figure 5). As evidenced, CNO induced a decrease in the time immobile in hM4Di-expressing mice, thereby replicating the findings shown in Figure 2.

2. It would be appropriate to analyze figure 2a and 2d together, 2b and 2e, as well as 2c and 2f together using a two-way ANOVA. these aren’t really separate experiments. this is especially relevant given that it appears there may be an effect of virus (i.e. vehicle in 2a is lower than vehicle in 2d, etc). On this note, were there any tests for outliers performed? if not, why? it

appears that there are outliers in 2d and 2e. Moreover, were data tested for assumptions of normality? What about differences in variance between groups?

Response: Per the reviewer’s suggestion, we have now combined the data from the prior Figures: (1) 2a and 2d, (2) 2b and 2e, and (3) 2c and 2f. Therefore, these are integrated together for the current Figure 2b, 2c and 2d. Per the request of the eNeuro Senior Editor- In-Chief, we have now employed estimation statistics for these two group analyses. A table has been included with all of the estimation statistic results.

Minor concerns:

1. There are a few typos in the significance statement as well as scattered throughout the

manuscript; there are additional grammatical issues.

Response: Thank you for identifying these grammatical issues. We have now corrected these throughout the manuscript.

2. The authors show effects on sucrose consumption. It would be helpful to understand why the authors chose to not conduct a sucrose preference test as this is likely more valid for understand stress-induced anhedonia.

Response: Previous studies have examined sucrose consumption in the context of stress- induced anhedonia (Monleon et al., 1995). We were interested in studying reward related food consumption, given the role of the IPN in mediating satiety signals (Tuesta et al., 2019). As the mice are choosing to consume the sucrose pellets under full food conditions, their behavior also reflects a preference for sucrose under satiated conditions. This point has been clarified within the methods for test rationale.

3. The authors should include a citation/justify their use of the CNO dose. Moreover, this dose of CNO is more than sufficient to be back metabolized to clozapine and produce clozapine-like interoceptive stimulus effects in mice (Manvich et al., 2018). Given that clozapine has antagonist effects at 5-HT 2A/2C receptors, this needs to be discussed. Also why was subcutaneous administration of CNO selected?

Response: We have now included a more detailed rationale for the CNO dose and discussion of the back metabolism of CNO within the manuscript methods and discussion. Specifically, a previous study has found that a subcutaneous 3.5 mg/kg injection of CNO in mice increased free CNO levels in CSF and total brain concentration above EC50 for hM4Di from at least 15 to 60 minutes post injection (Jendryka et al., 2019), and moreover, the 3 mg/kg dose has been commonly used as a moderate dose in prior studies (typically range from 1-10 mg/kg in rodents). In regard to the potential effects of metabolized clozapine, we specifically selected a relatively low dose of CNO. However, we still considered the antagonistic effects of metabolized clozapine at 5HT2A/2C receptors, which is why we employed additional control conditions with CNO in the absence of hM4Di. Furthermore, the relative amount of back metabolized clozapine occurring within the ventral hippocampus at the dose provided

peripherally is unknown. Importantly, we found no effects of CNO alone in our control groups. Thus, the peripheral injection of CNO most likely does not produce interoceptive stimulus effects via the ventral hippocampus. However, it will be interesting for future studies to inject clozapine in the ventral hippocampus specifically to see if there are any differences in behavior. These points have been added to the discussion.

4. An experimental design flowchart would be helpful.

Response: Given that different cohorts of mice received different assessments, we have more clearly outlined the specific testing conducted with each experimental set of mice in the methods section.

5. A figure with nicotine and food self-administration across sessions would be ideal, including active, inactive, and infusions earned. Were lever presses different between groups? Were CS cue presentations paired with infusions? Also, motivation to seek nicotine is typically carried out with extinction and then reinstatement. Given the authors did not do this, this should be discussed as a limitation. What the authors are measuring is an index of nicotine reward, not motivation.

Response: We appreciate the reviewer’s thoughtful comments and have removed discussion of motivation from the manuscript. Since we are interested in the effects of vehicle or CNO on these behaviors, we have provided the data from every session in which these injections were administered. Per the reviewer’s request, we have now included the active and inactive lever presses for each experimental condition. All experimental groups exhibited a clear dissociation between the active and inactive lever presses (Figure 3); these data are consistent with the findings for the number of nicotine infusions. Food or nicotine delivery are paired with a cue light and 20-sec time out period; this has now been clearly denoted within the methods. In the drug addiction field, nicotine reinforcement is attributed to the mouse’s lever pressing behavior for intravenous nicotine self-administration. In our studies, we used a fixed-ratio 5 schedule of reinforcement, and as such, the mouse must press the lever 5 times to achieve a nicotine infusion, requiring a higher level of motivation to obtain the drug (as opposed to the more commonly used fixed ratio 1 schedule of reinforcement). In contrast, extinction/reinstatement is a measure of drug seeking, in the absence of the drug; this procedure does not measure nicotine intake, which was the focus of the current studies. Given prior findings revealing the role of the habenulo- interpeduncular pathway in nicotine reinforcement with the intravenous nicotine self- administration procedure, we hypothesized that this pathway may be downstream of the habenular inputs into the IPN. However, this was not the case, as no differences were found with nicotine intake using the intravenous nicotine self-administration procedure.

6. DOI also has actions at serotonin 2B receptors. Moreover, DOI is known to have hallucinogenic properties. Does DOI microinfused into the vHipp induce a hallucinogenic state? Discuss. On that note, the authors should discuss what the cellular effect of DOI in reversing DREADD-induced inhibition of the IPN-vHipp may be. For example, DOI has been shown to alter structural plasticity. Discuss.

Response: We appreciate this thoughtful point brought up by the reviewer. In our studies, DOI was used as a pharmacological tool to examine the circuit. We acknowledge that DOI has been shown to elicit hallucinogenic properties, however, these hallucinogenic properties are extremely difficult to assess in mice. Future studies would be interesting to assess the specific cellular effect of DOI in reversing DREADD-induced inhibition. Given that both DOI and serotonin result in activation of the serotonin 2B receptors and that structural plasticity may be induced with such activation (as previously demonstrated in the amygdala, Chen et al., 2003), it is possible that the downstream effects on behavior could include changes in structural plasticity. These interesting points have been added to the manuscript.

7. In line 369, the authors refer to the DREADD construct as anterograde. This is confusing, as it makes it sound like a tracer. Reword.

Response: We have removed the word anterograde from the description of the DREADD

construct.

8. The time spent in the center of the open field (related to figure 3) should be included as a supplement given that the data was analyzed. Alternatively, provide the mean +/-SEM in the text as opposed to a statistical analysis.

Response: Extended Data figures have been added for the time spent in the center of the open field for both locomotor studies. Estimation statistical reporting in the results and a corresponding statistical table have been included.

9. The authors indicate on line 419 that their effect on food consumption is consistent with previous research. Cite that research, please.

Response: These references have now been provided.

10. figure 4 - some indication of the number of mcherry+ cells analyzed should be included to give the reader a better idea of what the denominator was and whether this was different between groups. Also, did the CNO manipulation decrease cfos in non-dreadd expressing neurons?

Response: The total number of mCherry+ cells quantified for the vehicle- and CNO-injected groups was 54 and 50, respectively. This information has been added to the manuscript. We did not examine cfos expression in non-DREADD expressing neurons, as this was not the focus of the current studies.

Reviewer2

The authors dissect out a serotonergic pathway from the interpeduncular nucleus to the ventral

hippocampus using intersectional genetic strategies and chemogenetic approaches to demonstrate that this pathway plays an important role in active coping, escape behaviors, as well as influencing sucrose consumption and motivation for food-related rewards. They illustrate that these effects of modulation of the IPN-vHippocampal 5-HT pathway can be overridden by the administration of the 5-HT2A/c receptor agonist DOI via cannulae directly

into the ventral hippocampus. The studies are of interest to the field and suggest a rather different role for this 5-HT pathway, contrasting from the existent literature. However, there are several points that do need to be addressed by the authors.

Specific points

1. The authors may want to illustrate the precise genetic strategy and mode of administration of either the DREADD ligand or the 5-HT2A/C agonist above each figure in an illustrated schematic to make it easier for the reader.

Response: Thank you for this suggestion to clarify the genetic strategy for the reader. We have now included illustrations indicating the viral vector injection strategy for each brain region. The same approach was employed for Figures 2, 3 and 4, so the Figure 2 schematics were referenced in the figure legends of Figures 3 and 4. Given the cannulations and site specific injections for the experiments in Figure 5, we included additional illustrations for this part of the study.

2. The study was carried out in singly housed mice and that opens up the question as to whether the same effects would be noted in group housed animals that do not have an overlay of isolation stress.

Response: This is indeed an important point. Our experimental design was to single house the mice to induce a mild stressful state, as noted in the methods, which would thereby allow for us to detect an increase or decrease in the behavioral tasks. We proposed that if the animals were grouped housed, a ceiling effect in their behavior may be imposed. However, group housing conditions will certainly be of interest for future studies. This point has now been addressed in the discussion.

3. Does activation of the IPN-vHippocampal 5-HT pathway modulate escape behaviors, either reducing active coping and toggling to passive coping strategies or resulting in reduction overall in escape behaviors?

Response: This would be an interesting study to perform as further investigation of the function of this pathway. We have added this point to the discussion.

4. Would delivering a 5-HT2A or 2C antagonost into the ventral hippocampus override the effects of activation of the serotonergic IPN-vhippocampal pathway?

Response: This is an interesting consideration. Given the localization of the receptors both on the pre- and post-synaptic membranes, such studies may lead to intriguing findings. However, since our aim was to validate the effect of inhibition of the IPN-vHipp pathway as being consistent with serotonergic signaling, we administered the agonist during pathway inhibition. However, future studies should certainly investigate this possibility.

5. Does delivering DOI into the ventral hippocampus per se influence overall locomotion?

Response: We appreciate this valuable suggestion from the reviewer to strengthen our conclusions. We have now included an additional experiment to examine the effects of DOl in both CNO and Vehicle injected mice. Consistent with our prior conclusions, we found no significant differences in locomotion under any of these experimental conditions (Figure Sd).
