# Supplementary figures and images for: The Interpeduncular-Ventral Hippocampus Pathway Mediates Active Stress Coping and Natural Reward
Source: eNeuro. 2020 Nov 16;7(6):ENEURO.0191-20.2020. doi: 10.1523/ENEURO.0191-20.2020 (PMC7688303; doi:10.1523/ENEURO.0191-20.2020)

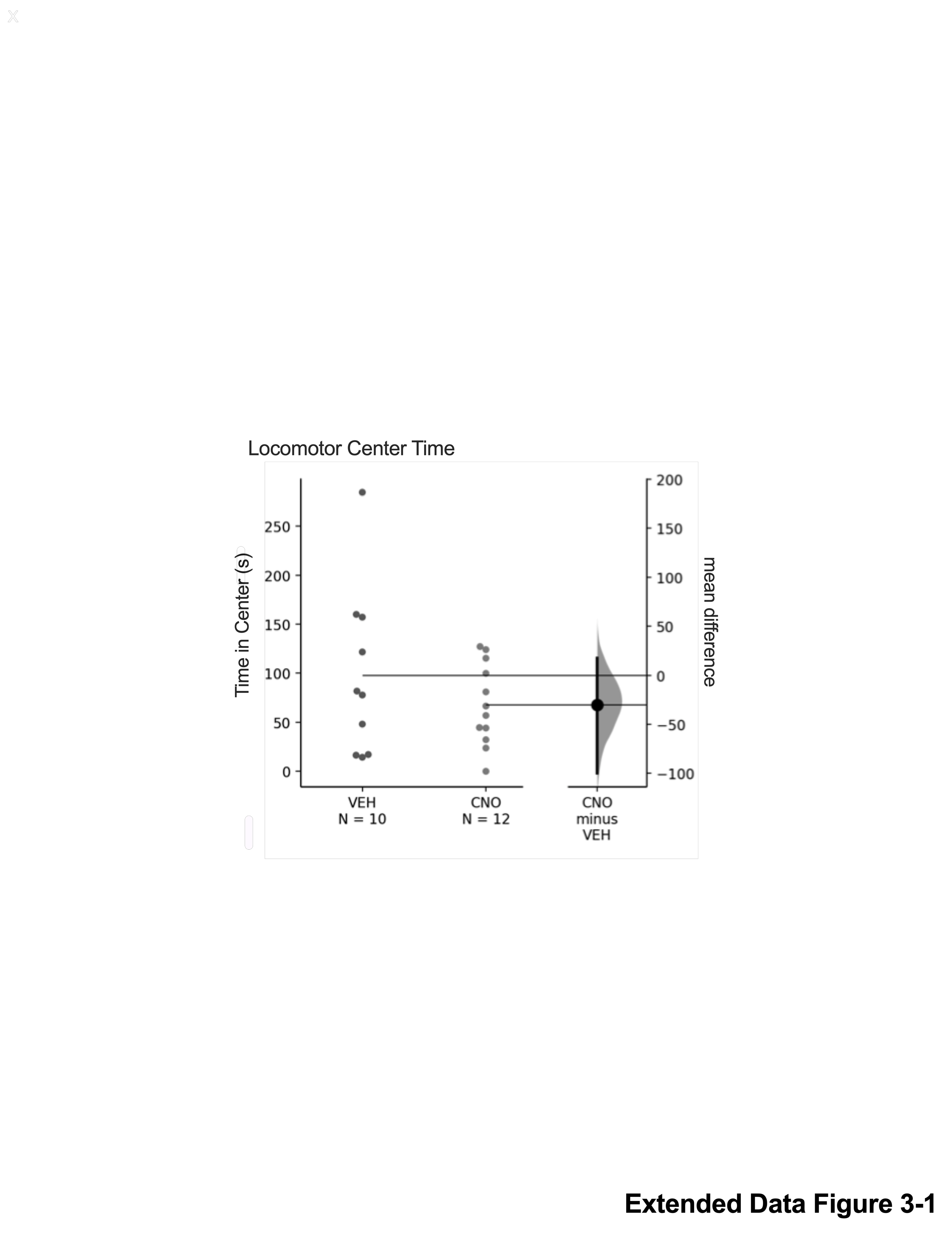

Supplement: Extended Data Figure 3-1 — Mice expressing hM4Di DREADD in the IPN-vHipp pathway did not differ in time spent in the center of an open field following vehicle (VEH) or CNO injection. Download Figure 3-1, TIF file. [file enu-eN-NWR-0191-20-s03.tif]

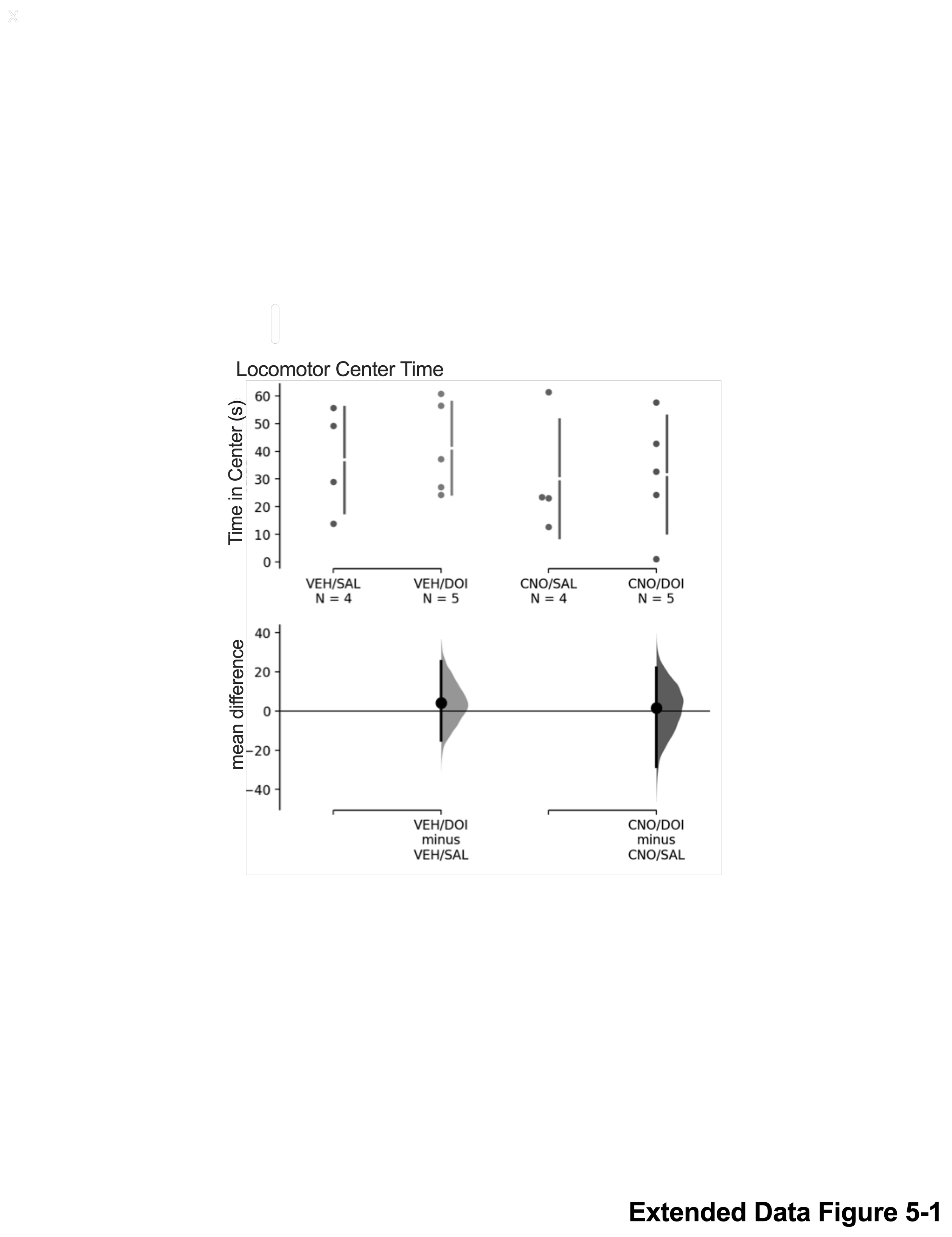

Supplement: Extended Data Figure 5-1 — Mice expressing hM4Di in the IPN-vHipp were injected with CNO or vehicle (VEH) subcutaneously, and then microinjected with DOI or Saline (SAL) directly into the vHipp via dual guide cannula. No differences were found in the time spent in the center of the open field among groups following treatment. Download Figure 5-1, TIF file. [file enu-eN-NWR-0191-20-s01.tif]
